# Establishment of quantitative and consistent in vitro skeletal muscle pathological models of myotonic dystrophy type 1 using patient-derived iPSCs

**DOI:** 10.1038/s41598-022-26614-z

**Published:** 2023-01-11

**Authors:** Ryu Kawada, Tatsuya Jonouchi, Akihiro Kagita, Masae Sato, Akitsu Hotta, Hidetoshi Sakurai

**Affiliations:** 1 Department of Clinical Application, Center for iPS Cell Research and Application (CiRA), Kyoto University, Kyoto, 606-8507 Japan; 2 Discovery Research Laboratories, Taisho Pharmaceutical Co., Ltd., Saitama, 331-9530 Japan

**Keywords:** Drug screening, Pharmacology, Mechanisms of disease, Neuromuscular disease

## Abstract

Myotonic dystrophy type 1 (DM1) is caused by expanded CTG repeats (CTGexp) in the dystrophia myotonica protein kinase (*DMPK*) gene, and the transcription products, expanded CUG repeats, sequester muscleblind like splicing regulator 1 (MBNL1), resulting in the nuclear MBNL1 aggregation in the DM1 cells. Loss of MBNL1 function is the pivotal mechanism underlying the pathogenesis of DM1. To develop therapeutics for DM1, proper human in vitro models based on the pathologic mechanism of DM1 are required. In this study, we established robust in vitro skeletal muscle cell models of DM1 with patient-derived induced pluripotent stem cells (iPSCs) using the MyoD1-induced system and iPSCs-derived muscle stem cell (iMuSC) differentiation system. Our newly established DM1 models enable simple quantitative evaluation of nuclear MBNL1 aggregation and the downstream splicing defects. Quantitative analyses using the MyoD1-induced myotubes showed that CTGexp-deleted DM1 skeletal myotubes exhibited a reversal of MBNL1-related pathologies, and antisense oligonucleotide treatment recovered these disease phenotypes in the DM1-iPSCs-derived myotubes. Furthermore, iMuSC-derived myotubes exhibited higher maturity than the MyoD1-induced myotubes, which enabled us to recapitulate the SERCA1 splicing defect in the DM1-iMuSC-derived myotubes. Our quantitative and reproducible in vitro models for DM1 established using human iPSCs are promising for drug discovery against DM1.

## Introduction

Myotonic dystrophy type 1 (DM1) is a multisystemic disorder as mainly manifested by myotonia, progressive muscle weakness, cardiac conduction defects, and cognitive impairment^1^. DM1 is caused by expanded CTG repeats (CTGexp) in the 3’ untranslated region of the dystrophia myotonica-protein kinase (*DMPK*) gene. This mutated expansion results in the production of foci of expanded CUG repeats (CUGexp) in the nuclei^2^. CUGexp cause deregulation of alternative splicing regulators such as muscleblind like splicing regulator 1 (MBNL1) and the CUG-binding protein, ELAV-like family member 1 (CELF1)^3^. In particular, MBNL1 is sequestered by CUGexp, leading to the formation of intranuclear MBNL1 aggregates in the DM1 cells^4^. The intranuclear MBNL1 aggregation is associated with foci of CUGexp^5^. Importantly, functional loss of MBNL1 results in numerous alternative splicing defects and represents the central pathogenetic mechanism of DM1^6,7^.

Although DM1 is the most common form of muscular dystrophy in adults, there is currently no curative treatment. Several mouse models of DM1 have been developed and studied for research on DM1, whereas the most common of which is the HSA^LR^ DM1 mouse expressing 250 CTG repeats in a human skeletal actin (ACTA1) transgene^8^, where is different from the genuine mutation locus in DM1 patients. The DMSXL mouse carrying > 1000 CTG repeats in the human DMPK transgene exhibits milder splicing defects in the skeletal muscles and heart than those observed in DM1 patients^9^. Although other in vivo models such as DM200 mice are reported to develop severe mis-splicing^10^, there is a definite need for human in vitro models accurately recapitulating the DM1-related disease features in view of the understanding of the pathogenetic mechanisms of DM1 and the throughput for drug screening. In particular, patient-derived cells could have the advantage of studying the wide phenotype spectrum of DM1 patients with a different number of CTG repeats. Many of in vitro models of DM1 have been established using DM1 patient-derived primary cells and their immortalized cells^11^. To advance research on DM1, MyoD1-induced primary dermal fibroblasts and primary myoblasts derived from DM1 patients are helpful and have been well studied^4,12,13^. However, for drug screening, preparation of sufficient cell stocks from the biopsy samples is not feasible due to the limited proliferative abilities of these primary cells.

For in vitro disease modeling, human induced pluripotent stem cells (hiPSCs) provide powerful advantages. hiPSCs can be generated from patient-derived somatic cells harboring the mutation, and show unlimited proliferative capacity^14^. Furthermore, hiPSCs have the potential to differentiate into a wide spectrum of cell types in systemic tissues^15^. The pluripotency of hiPSCs is a clear advantage to study DM1, which is a multisystemic disorder affecting multiple tissues, e.g., skeletal muscles, heart, and brain. We previously reported two methods to induce differentiation of hiPSCs into skeletal muscle cells. The first one, the doxycycline-induced MyoD1 overexpression system, has been applied to disease modeling for several muscular dystrophies^16–18^ and been proven to be valuable for the investigation of CTGexp instability in DM1 cells^19^. The other is the iPSC-derived muscle stem cell (iMuSC) induction system, in which hiPSCs are induced to differentiate into fetal muscle stem cells (MuSCs) by recapitulating myogenesis^20^. The iMuSC shows a high regeneration potential in vivo and high myogenic capacity in vitro^20–22^.

To advance DM1 therapeutics, quantitative methods for analyzing molecular DM1-related phenotypes, as well as reproducible in vitro disease models are essential. In situ hybridization is well established as a method for the evaluation of CUGexp foci ^23^. Indeed, Maury et al*.*^24^ reported DM1 drug screening on the basis of evaluation of CUGexp foci. However, there is still a need for a quantitative method to evaluate nuclear MBNL1 aggregation, which is the pivotal molecular mechanism in the pathogenesis of DM1.

Herein, we report novel in vitro skeletal muscle cell models of DM1 differentiated from three different patient-derived iPSCs by MyoD1-induced myogenic differentiation and in vitro iMuSC differentiation. The in vitro DM1 models established thus successfully recapitulated two important DM1-related phenotypes, namely, nuclear MBNL1 aggregation and the downstream alternative splicing defects, as validated by newly established quantitative analyses. In the model established by MyoD1-induced differentiation, we modified the previous MyoD1 overexpression method, in which replating and electrical field stimulation (EFS) were performed for myogenic maturation^18^, to establish a simpler protocol for efficient induction of hiPSCs to differentiate into skeletal myotubes by the addition of cytosine β-d-arabinofuranoside (Ara-C) to prevent outgrowth of non-myogenic cells. DM1-associated splicing defects of *DMD* exon 78 and *BIN1* exon 11 were clearly detected by the modified protocol, and treatment with the morpholino antisense oligonucleotide (ASO), CAG25, significantly restored nuclear MBNL1 aggregation and the splicing defects. Furthermore, the mature iMuSC-derived myotubes enabled recapitulation of a major functional molecule in the skeletal muscles, namely sarcoplasmic/endoplasmic reticulum (SR) calcium-ATPase 1 (SERCA1) mis-splicing, in the DM1 cells. Our results suggest that the in vitro skeletal muscle cell models of DM1 obtained by differentiation of hiPSCs are valuable to study DM1 pathology in the skeletal muscles and facilitate drug discovery for the treatment of DM1.

## Results

### Nuclear MBNL1 aggregation in the myotubes differentiated from MyoD-DM1-hiPSCs

**Figure 1:**
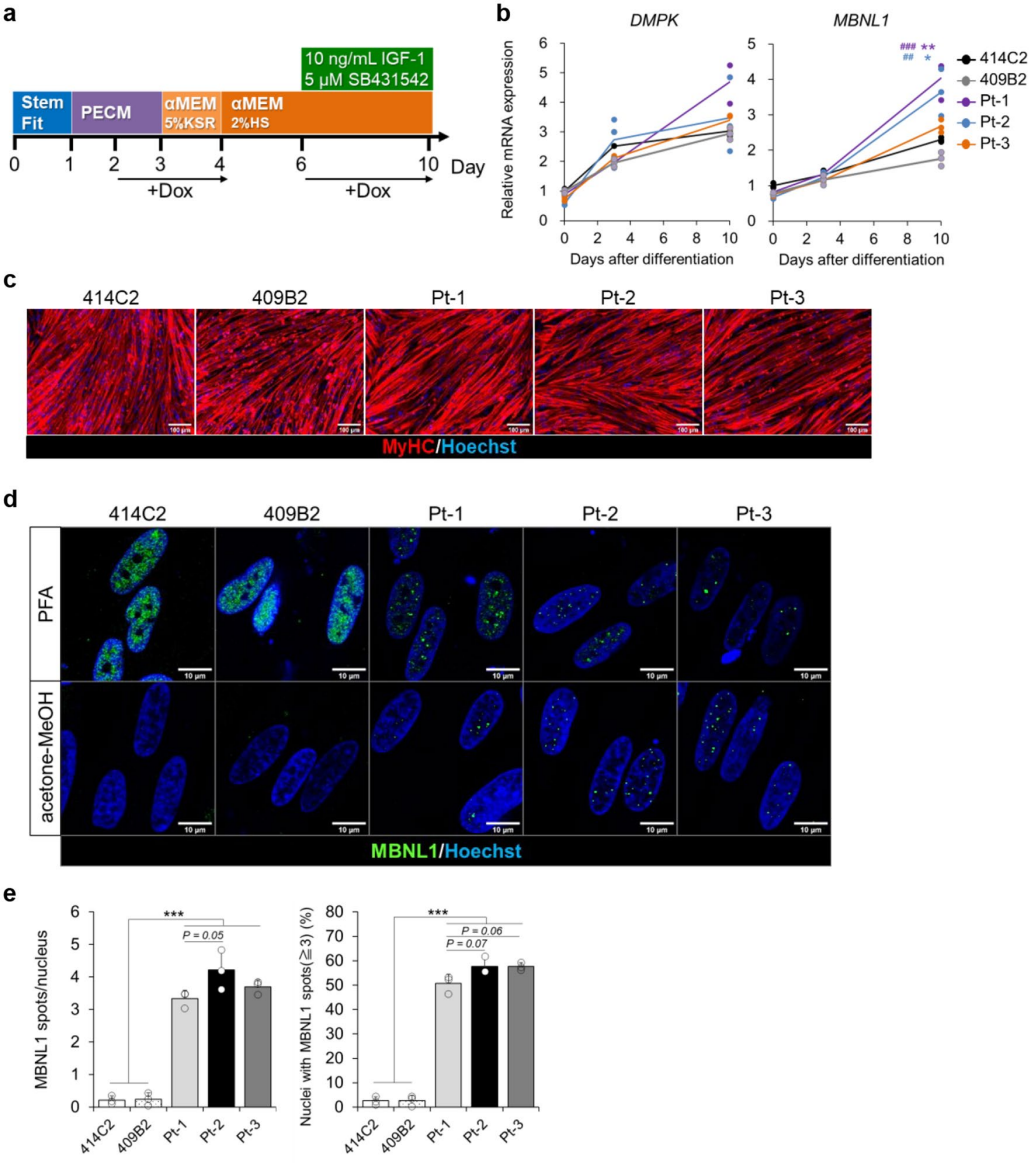
Nuclear MBNL1 aggregation in the myotubes differentiated from MyoD-DM1-hiPSCs. (**a**) A schematic protocol of the MyoD1-induced differentiation for 10 days, with reference to our previous report^18^. (**b**) Time-course of mRNA expressions of the DM1-related genes, *DMPK* and *MBNL1* by RT-qPCR analyses on day 0, day 3 and day 10 of differentiation. The gene expressions are indicated by relative values to 414C2 control on day 0. The data represent the means of three independent experiments and were analyzed by one-way ANOVA followed by Tukey’s test (*P < 0.05, **P < 0.01, vs. 414C2 control and ## *P* < 0.01, ### *P* < 0.001, vs. 409B2 control). (**c**) The representative immunohistochemistry images for MyHC and Hoechst on day 10. Scale bars, 100 μm. (**d** and **e**) Evaluation of nuclear MBNL1 aggregation on day 10. (**d**) Representative immunohistochemistry images after PFA or acetone-MeOH fixation for MBNL1 and Hoechst. Scale bars, 10 μm. (**e**) Quantitative analyses of the images of MBNL1 immunostaining after acetone-MeOH fixation using the ImageJ software. The nuclear regions were detected by Hoechst counterstaining. Data represent the means + SD of three independent experiments and were analyzed by one-way ANOVA followed by Tukey’s test (***P < 0.001).

Previously, we reported a myogenic differentiation method using MyoD-hiPSCs^16,18^. In this study, five MyoD-hiPSC lines (two control-hiPSC lines: 414C2, 409B2 and three DM1-hiPSC lines: Patient-1 (Pt-1), Patient-2 (Pt-2) and Patient-3 (Pt-3)) were induced to differentiate into myocytes to recapitulate DM1 phenotypes of skeletal muscles according to a previously reported method^18^ (Fig. 1a). The cells expressed *DMPK* and *MBNL1*, as well as the myogenic markers, *MYOD1* and *MYOGENIN*, and the embryonic skeletal muscle marker, *MYH3,* after differentiation (Fig. 1b and Supplementary Fig. S1a). In addition, robust myosin heavy chain (MyHC) expression was also confirmed by immunocytochemistry on differentiation day 10 (Fig. 1c). Then, we performed MBNL1 immunostaining on day 10 to investigate the distribution of MBNL1 in the hiPSC-derived myotubes for detecting the phenotype of DM1. By the conventional fixation method using PFA, the control (Cntl) myotubes showed diffuse distribution of MBNL1 throughout the nuclei, while in contrast, the DM1 myotubes exhibited spot-like nuclear distribution (Fig. 1d). The spot-like MBNL1 signals were colocalized with CUGexp foci in the DM1 myotubes indicated by fluorescence in situ hybridization (FISH)-immunohistochemistry (Supplementary Fig. S1b). However, quantitative determination by automatic image analysis was difficult by this method, because the Cntl myotubes displayed MBNL1-positive signals of high intensity. To overcome this problem, we attempted another fixation method using acetone-methanol (MeOH) by referring to a previous report^25^. In this method, almost all of the signals from soluble MBNL1 in the Cntl myotubes disappeared, whereas the signals from the aggregated insoluble MBNL1 interacting with CUGexp in the DM1 myotubes were observed (Fig. 1d and Supplementary Fig. S1b), with automatic quantitative analysis by counting of the spot-like signals with a software. The results revealed significant increases in the number of MBNL1 aggregates per nucleus and percentage of nuclei showing MBNL1 aggregation was significantly increased in the DM1 myotubes on day 10 (Fig. 1e). Furthermore, the number of nuclear MBNL1 aggregates in the DM1 cells increased in a differentiation-time-dependent manner according to the upregulation of *DMPK* and *MBNL1* (Supplementary Fig. S2a–c). These data indicate the establishment of a quantitative method for counting nuclear MBNL1 aggregation of MyoD-hiPSCs at day 10 differentiation. In this study, when conducting MBNL immunostaining, we always used two fixation methods (PFA and acetone-MeOH) and the latter one was used for quantitative analysis.

### Splicing defects in the myotubes differentiated from MyoD-DM1-hiPSCs

**Figure 2:**
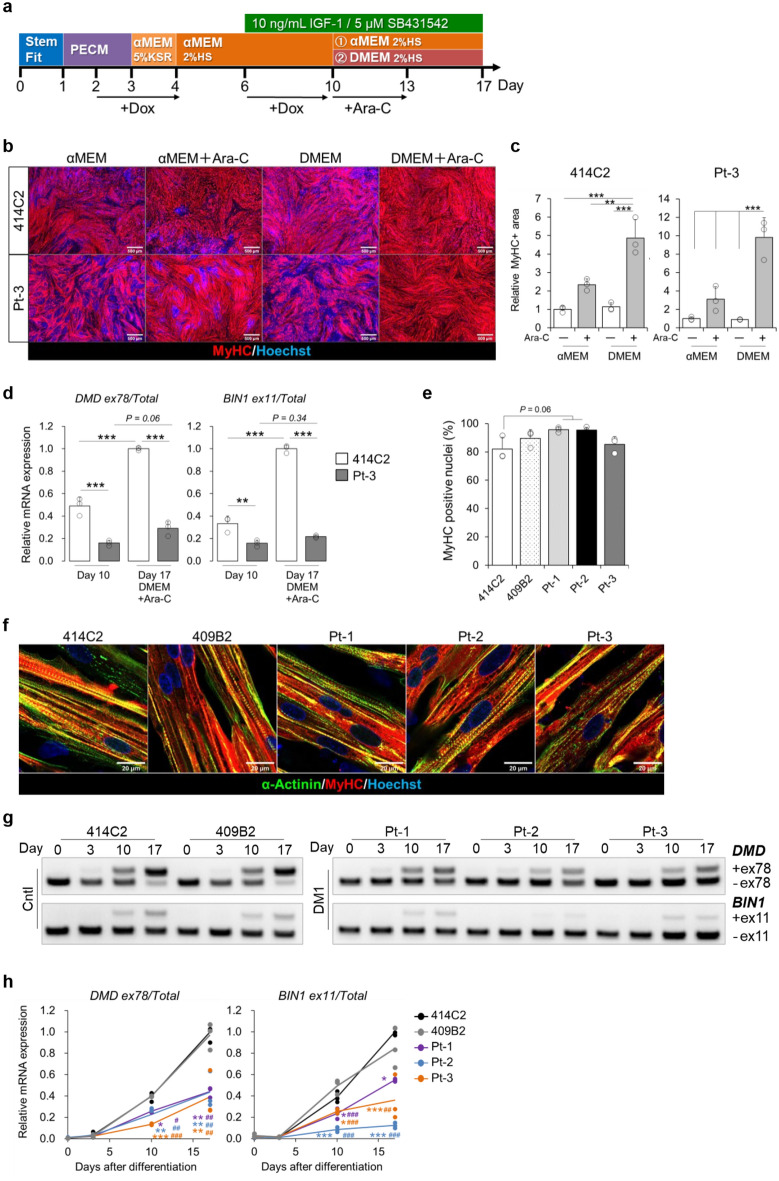
Splicing defects in the myotubes differentiated from MyoD-DM1-hiPSCs using the newly modified protocol for the MyoD1-induced system. (**a**) A schematic protocol of the MyoD1-induced differentiation for 17 days. Ara-C was added to the culture for three days from day 10 and the cells were cultured in αMEM + 2% HS or DMEM + 2%HS from day 10. (**b** and **c**) Evaluation of MyoD1-induced myogenic differentiation in the 414C2 and Pt-3 myotubes on day 17. (**b**) Representative immunohistochemistry images for MyHC and Hoechst. Scale bars, 500 μm. (**c**) Quantitative analysis of the MyHC-positive area with the BZ-X analyzer software. Data represent the means + SD of three independent experiments and were analyzed by two-way ANOVA followed by Tukey’s test (**P < 0.01, ***P < 0.001). (**d**) Quantitative analyses of alternative splicing for *DMD* and *BIN1* by RT-qPCR analyses on day 10 and day 17. The mRNA expressions of *DMD* exon 78 and *BIN1* exon 11 were normalized to the total expression level of each target gene. Data represent the means + SD of three independent experiments and were analyzed by two-way ANOVA followed by Tukey’s test (**P < 0.01, ***P < 0.001). (**e**) The differentiation efficiency calculated by the percentage of Hoechst-positive counts (nuclei) in the MyHC-positive area in the MyHC immunostained samples. Data represent the means + SD of three independent experiments and were analyzed by one-way ANOVA followed by Tukey’s test. (**f**) Representative immunohistochemistry images for α-Actinin, MyHC and Hoechst on day 17. Scale bars, 20 μm. (**g** and **h**) Evaluation of alternative splicing of *DMD* and *BIN1* on day 0, day 3, day 10, and day 17. (**g**) Representative images obtained by gel-based RT-PCR. The target exons are indicated on the right. (**h**) The results of quantitative analyses by RT-qPCR. The gene expressions are indicated by relative values to 414C2 control on day 17. Data represent the means of three independent experiments and were analyzed by one-way ANOVA followed by Tukey’s test (*P < 0.05, **P < 0.01, ***P < 0.001, vs. 414C2 control and # *P* < 0.05, ## *P* < 0.01, ### *P* < 0.001, vs. 409B2 control).

To evaluate DM1-associated and MBNL1-dependent splicing events in our MyoD1-induced system, alternative splicing of *DMD* and *BIN1* transcripts was assessed by RT-PCR^26,27^. Although the expression of *BIN1* was confirmed by RT-PCR, mis-splicing of *BIN1* was undetectable in the DM1 myotubes derived from Pt-3 (Supplementary Fig. S3a). The result seems to have been caused by immaturity of the MyoD-hiPSC-derived myotubes on day 10. Therefore, the culture period was extended until day 17 to allow for myogenic maturation, but this also led to unexpected gradual increase of non-myogenic and proliferative cell populations. To eliminate these populations, we investigated the effect of Ara-C on the MyoD-hiPSC differentiation system. Ara-C is a nucleoside analog which interferes with DNA replication, causing apoptosis of proliferating cells^28^. Therefore, Ara-C has been used for obtaining pure neuronal cell cultures^29^ and also myotube cultures^30^. To investigate the optimal culture conditions for maturation, we also compared the media, αMEM and DMEM (Fig. 2a). After adding Ara-C to 414C2 and DM1 cells from Pt-3 for three days starting on day 10, MyHC immunostaining was performed on day 17 to evaluate myogenic differentiation. The results revealed larger MyHC-positive areas in the Ara-C treated cells than in the non-Ara-C-treated cell cultures (Fig. 2b and c), and the culture conditions of DMEM + Ara-C were most effective for obtaining pure myotube cultures from both cell lines. Accordingly, the mRNA expressions of the neonatal marker, *MYH8*, and the mature alternative splicing forms, *DMD* exon 78 and *BIN1* exon 11, were highest in the DMEM + Ara-C culture (Supplementary Fig. S3b and S3c). Importantly, large amounts of *DMD* exon 78 and *BIN1* exon 11 inclusions were measured in the conditioned medium on day 17 as compared with that on day 10 in the previous protocol (Fig. 2d). This modified protocol resulted in a big difference in the amounts of *DMD* exon 78 and *BIN1* exon 11 inclusions between the Cntl and DM1 myotubes (Fig. 2d). Therefore, we applied the new protocol to the other three cell lines too (409B2, Pt-1 and Pt-2). In these cultures also, the MyHC-positive areas in the Ara-C treated cells were larger than those in the non-Ara-C-treated cells (Supplementary Fig. S3d and S3e). In the conditioned medium of DMEM + Ara-C on day 17, the percentages of MyHC-positive nuclei (differentiation efficiency) in all five cell lines were more than 80% (Fig. 2e) and these myotubes similarly possessed a sarcomere-like pattern of α-actinin (Fig. 2f). These data indicate the applicability of this new protocol for maturation of MyoD-hiPSC-derived myotubes. Next, time-course experiments were conducted using the five MyoD-hiPSC lines, and all the cells gradually changed their morphology. They showed spindle-shaped morphology on day 10, and this morphology was maintained until day 17 (Supplementary Fig. S4a). RT-qPCR was performed to evaluate the gene expressions. All the cells showed dramatic decrease in the expressions of the pluripotency markers, *OCT-3/4*, *NANOG*, and *SOX2* (Supplementary Fig. S4b), and in contrast, increase in the expressions of *DMPK* and *MBNL1* (Supplementary Fig. S4c), as well as of *MYOD1*, *MYOGENIN*, *MYH3*, and *MYH8* after differentiation (Supplementary Fig. S4d). In addition, alternative splicing of *DMD* and *BIN1* transcripts was evaluated by gel-based RT-PCR for qualitative assessment and RT-qPCR for quantitative assessment. The results revealed a shift of these splicing patterns from the fetal to adult splice isoforms during myogenic maturation in the Cntl cell lines (Fig. 2g and h); by contrast, the DM1 cell lines predominantly expressed the fetal splice isoforms even after maturation. Thus, clear differences in the alternative splicing patterns were observed between the Cntl and DM1 cells after differentiation, especially on day 17 (Fig. 2g and h). These data indicate that splicing defects in the MyoD-DM1-hiPSC-derived myotubes can be identified by the modified culture protocol and that analysis on day 17 would be appropriate for evaluation of the drug efficacy.

### Reversal of the DM1 phenotypes in CTGexp-deleted DM1-hiPSCs

**Figure 3:**
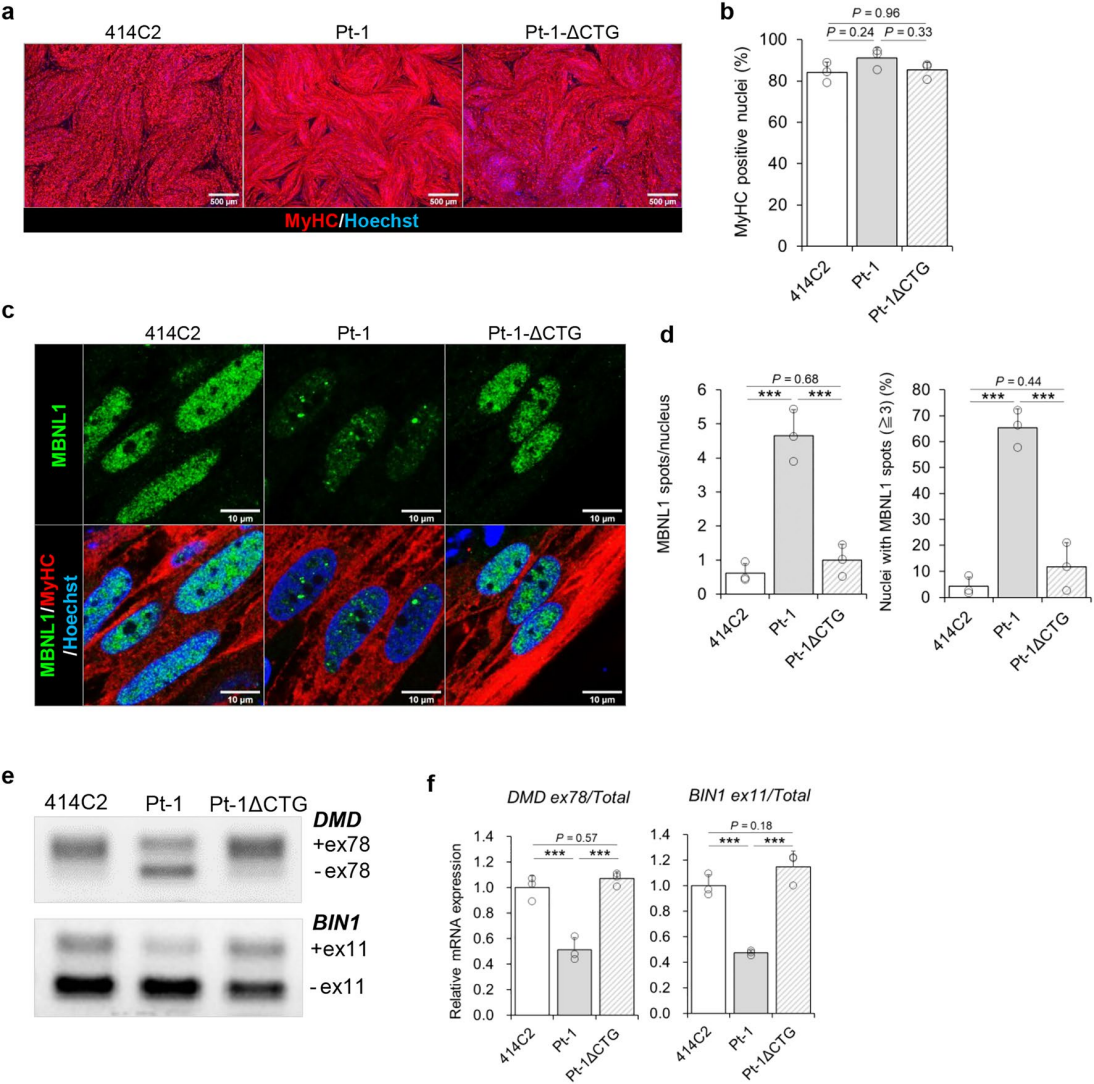
Reversal of the DM1 phenotypes after deletion of CTGexp in the MyoD-DM1-hiPSCs by CRISPR-Cas9. (**a** and **b**) Evaluation of MyoD1-induced myogenic differentiation efficiency in the 414C2, Pt-1 and Pt-1ΔCTG myotubes on day 17. (**a**) Representative immunohistochemistry images for MyHC and Hoechst. Scale bars, 500 μm. (**b**) Quantitative analyses of the MyHC-positive area with the BZ-X analyzer software. Data represent the means + SD of three independent experiments and were analyzed by one-way ANOVA followed by Tukey’s test. (**c** and **d**) Evaluation of nuclear MBNL1 aggregation on day 10. (**c**) Representative immunohistochemistry images after PFA fixation for MBNL1, MyHC and Hoechst. Scale bars, 10 μm. (**d**) Quantitative analyses of the MBNL1 immunostained samples after acetone-MeOH fixation using an ImageJ software. The nuclear regions were detected by Hoechst counterstaining. Data represent the means of three independent experiments and were analyzed by one-way ANOVA followed by Tukey’s test (***P < 0.001). (**e** and **f**) Evaluation of alternative splicing of *DMD* and *BIN1* on day 17. (**e**) Representative images obtained by gel-based RT-PCR. The target exons are indicated on the right. (**f**) The results of quantitative analyses by RT-qPCR. Data represent the means + SD of three independent experiments and were analyzed by one-way ANOVA followed by Tukey’s test (***P < 0.001).

To confirm that these DM1-associated phenotypes were caused by CTGexp of the *DMPK* gene, we generated isogenic control for DM1-hiPSCs from Pt-1 by the CRISPR-Cas9 method utilizing two sgRNAs to cut out the 5'-side and 3'-side of the CTG repeats^31^. After electroporation of Cas9 and two sgRNAs into the iPSCs, we successfully established one CTGexp-deleted subclone (Pt-1ΔCTG) (Supplementary Fig. S5a). By using 414C2 (healthy control), Pt-1 (DM1 patient) and Pt-1ΔCTG (genome-edited) iPSC lines, we first assessed the myogenic differentiation efficiency by immunocytochemistry for MyHC. All three cell lines showed similar and efficient differentiation into myotubes (Fig. 3a), and the efficiency of generation of the MyHC-positive myogenic cells was more than 80% for all three lines (Fig. 3b). Next, nuclear MBNL1 aggregation was investigated by immunocytochemistry. The results revealed spot-like distribution of MBNL1 protein in the cells from Pt-1, but in contrast, diffuse distribution of MBNL1 throughout the nuclei in the Pt-1ΔCTG cells, similar to the case in 414C2 cells (Fig. 3c). Accordingly, quantitative analysis indicated complete abrogation of nuclear MBNL1 aggregation in the Pt-1ΔCTG line, comparable to that in the 414C2 cells (Fig. 3d and Supplementary Fig. S5c). Finally, alternative splicing for *DMD* and *BIN1* transcripts was evaluated in these cells. The results revealed complete reversal of the splicing defects in the Pt-1ΔCTG cells as compared with the Pt-1 cells (Fig. 3e, f and Supplementary Fig. S5d), which was consistent with the diffuse distribution of MBNL1 in the Pt-1ΔCTG cells. The expressions of *MYH8*, *DMPK* and *MBNL1* were comparable between Pt-1 and Pt-1ΔCTG cells (Supplementary Fig. S5e), which suggested that the reversal of the phenotype did not arise from the difference in their maturity. These data indicate that nuclear MBNL1 aggregation and splicing defects were caused by the CTGexp and the removal of the CTGexp by CRISPR-Cas9 genome editing successfully recovered the DM1 phenotypes.

### Evaluation of drug efficacy

**Figure 4:**
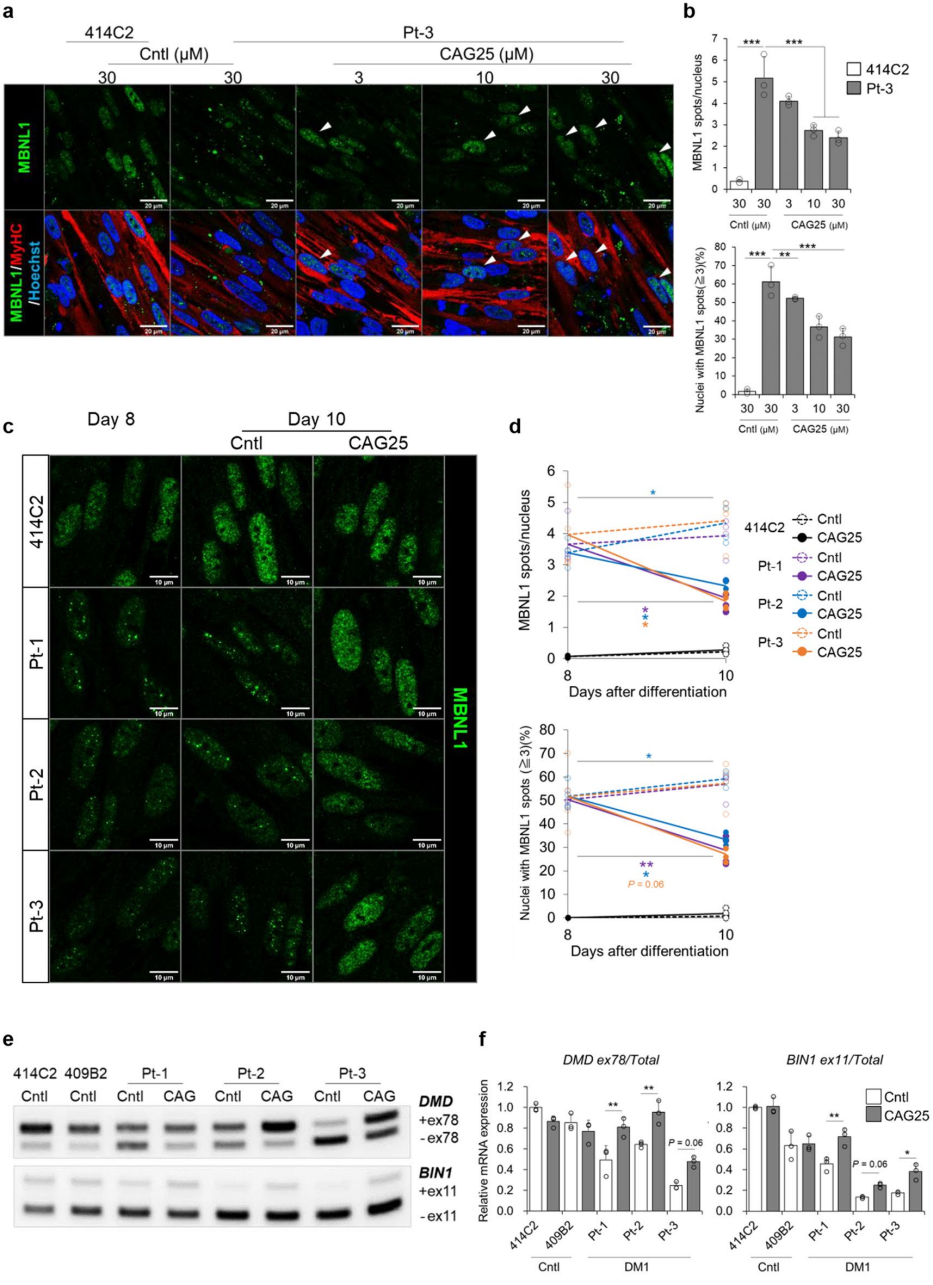
Recovery of the DM1 phenotypes by CAG25 treatment in the MyoD1-induced system. (**a** and** b**) Evaluation of nuclear MBNL1 aggregation after two days of CAG25 treatment in Pt-3 myotubes on day 10. (**a**) Representative immunohistochemistry images after PFA fixation for MBNL1, MyHC and Hoechst. Diffuse distribution of MBNL1 was observed in a proportion of the CAG25-treated Pt-3 nuclei (arrowheads). Scale bars, 20 μm. (**b**) Quantitative analyses of the MBNL1 immunostained samples after acetone-MeOH fixation using the ImageJ software. The nuclear regions were detected by Hoechst counterstaining. Data represent the means + SD of three independent experiments and were analyzed by two-way ANOVA followed by Tukey’s test (**P < 0.01, ***P < 0.001). (**c** and **d**) Evaluation of nuclear MBNL1 aggregation on day 8 and day 10. The cells were treated with CAG25 from day 8 to day 10. (** c**) Representative immunohistochemistry images after PFA fixation for MBNL1, MyHC and Hoechst. Scale bars, 10 μm. (**d**) Quantitative analyses of the MBNL1 immunostained samples after acetone-MeOH fixation. Data represent the means of three independent experiments and were analyzed by the paired t-test (*P < 0.05, **P < 0.01). (**e** and **f**) Evaluation of alternative splicing of *DMD* and *BIN1* after four days of CAG25 treatment on day 17. (**e**) Representative images obtained by gel-based RT-PCR. The target exons are indicated on the right. (**f**) The results of quantitative analyses by RT-qPCR. The gene expressions are indicated by relative values to 414C2 control treated with control oligonucleotide. Data represent the means + SD of three independent experiments and were analyzed by two-way ANOVA followed by Tukey’s test (*P < 0.05, **P < 0.01).

A previous report showed that the morpholino ASO, CAG25, binds CUGexp and blocks the interaction of CUGexp with MBNL1 in DM1 cells^32^. ASO treatment has been reported to result in an increase of functional MBNL1 and recovery of splicing defects^32^. Therefore, the efficacy of CAG25 was investigated in our in vitro DM1 model as a demonstration for evaluating a candidate drug efficacy. MyoD-DM1-hiPSC-derived myotubes were treated with CAG25 and evaluated for nuclear MBNL1 aggregation and splicing defects. After two days of CAG25 treatment, we observed diffuse distribution of MBNL1 in a proportion of DM1 nuclei from Pt-3 on day 10 (Fig. 4a, arrowheads). Quantitative analysis indicated that CAG25 treatment reduced nuclear MBNL1 aggregation in a dose-dependent manner (Fig. 4b and Supplementary Fig. S6a). Further analysis showed that the MBNL1 aggregates were increased in number only inside the nuclei in the DM1 myotubes, and that CAG25 treatment reduced them specifically without affecting the MBNL1 spots outside the nuclei (Supplementary Fig. S6b). Next, the efficacy of CAG25 was investigated in the other two DM1-hiPSC lines from Pt-1 and Pt-2. In this experiment, we performed immunocytochemistry for MBNL1 on day 8 and day 10. All the DM1 cell lines exhibited nuclear MBNL1 aggregation on day 8, and CAG25 treatment caused diffuse distribution of nuclear MBNL1 (Fig. 4c). Quantitative analysis showed that CAG25 treatment significantly reduced the nuclear MBNL1 aggregation seen in these DM1 myotubes from day 8 to day 10 (Fig. 4d and Supplementary Fig. S6c). These data indicate that nuclear MBNL1 aggregation in the DM1 myotubes is a reversible phenotype and that our in vitro DM1 model enables evaluation of the therapeutic effect of candidate drugs for DM1 on nuclear MBNL1 aggregation. Subsequently, alternative splicing of *DMD* and *BIN1* transcripts was investigated in the presence of CAG25. After CAG25 treatment, the DM1 myotubes derived from Pt-3 showed elevation of *DMD* exon 78 and *BIN1* exon 11 inclusions in a dose-dependent manner (Supplementary Fig. S7a–c). CAG25 treatment exerted no effect on the alternative splicing in the 414C2 cells (Supplementary Fig. S7d) or on the expressions of *MYH8*, *DMPK* and MBNL1 in the DM1 myotubes (Supplementary Fig. S7e). These data indicate that CAG25 treatment specifically affects DM1 cells and does not affect their maturity. Finally, we treated myotubes derived from the two Cntl-MyoD-hiPSC lines and three DM1-MyoD-hiPSC lines with CAG25 and evaluated alternative splicing in the cells. CAG25 treatment exclusively facilitated *DMD* exon 78 and *BIN1* exon 11 inclusions in these DM1 myotubes without exerting any effects on the expressions of *MYH8*, *DMPK* and *MBNL1* (Fig. 4e,f, Supplementary Fig. S7f. and S7g). These data validate the efficacy of genuine CAG25 in DM1-MyoD-hiPSCs and that this in vitro model is valuable for assessing the efficacy of a candidate drug.

### Acquisition of iMuSCs

**Figure 5:**
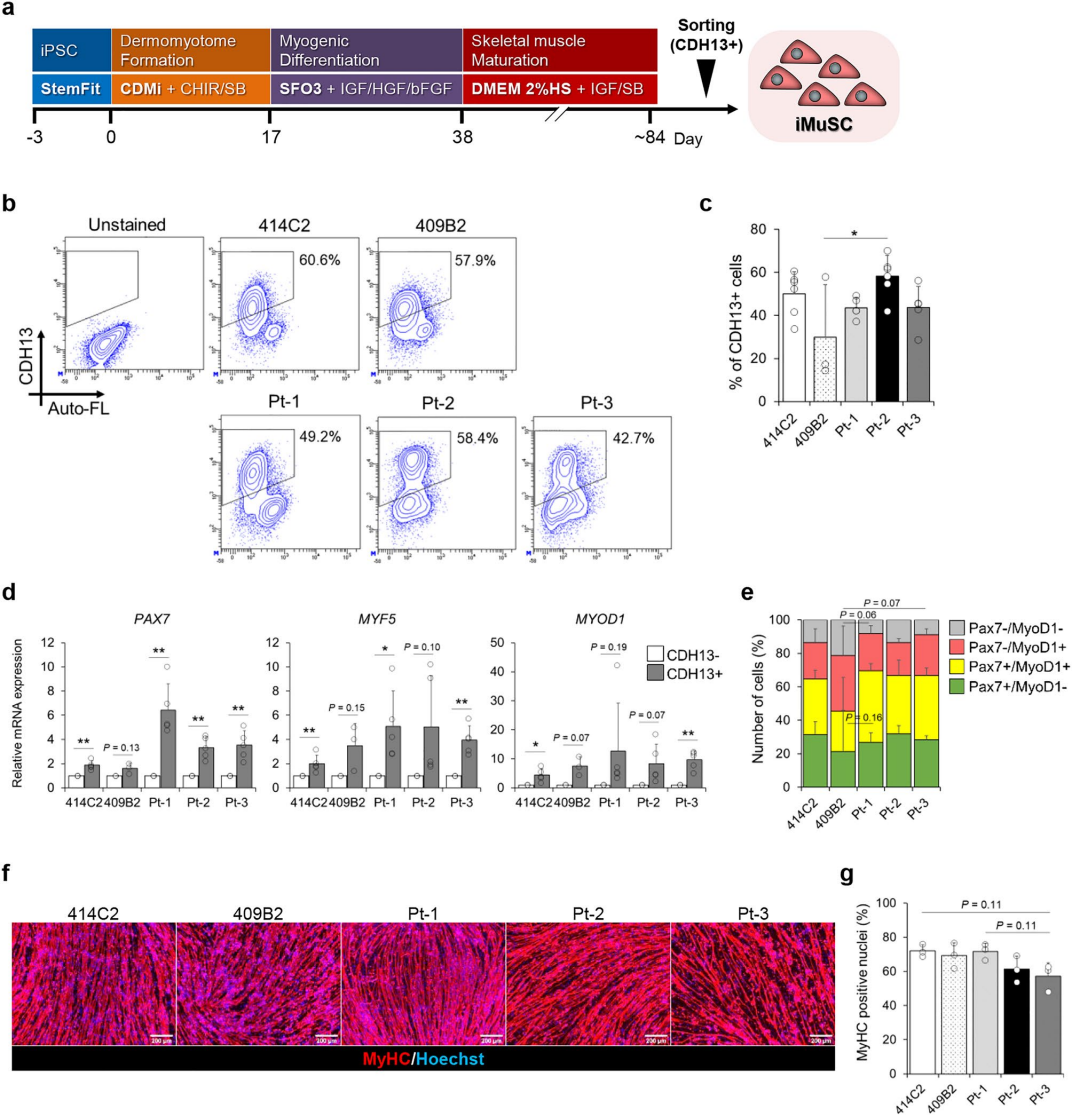
Myogenic induction of hiPSCs and evaluation of the myogenic capacity of CDH13-positive iMuSCs in vitro. (**a**) A schematic stepwise protocol for transgene-free differentiation for 13 weeks with reference to our previous report^20^. (**b** and **c**) Flow-cytometric analysis of CDH13 at week 12–13 of differentiation. (**b**) Representative images of flow-cytometry analysis of 414C2, 409B2, Pt-1, Pt-2, and Pt-3 cells for CDH13 expression. (**c**) Quantitative analysis of the CDH13-positive cells by flow cytometry. Data represent the means + SD of at least three independent experiments and were analyzed by one-way ANOVA followed by Tukey’s test (*P < 0.05). (**d**) mRNA expressions of the myogenic markers *PAX7*, *MYF5* and *MYOD1* in the sorted CDH13-negative and -positive cells by RT-qPCR analysis after sorting. The gene expressions are indicated by relative values to the CDH13-negative group in each cell line. Data represent the means + SD of at least three independent experiments and were analyzed by Welch’s t-test (*P < 0.05, **P < 0.01). (**e**) Quantitative analyses of Pax7 + , MyoD1 + , and Pax7 + MyoD1 + cells in the sorted CDH13-positive cells by immunocytochemistry. Data represent the means + SD of at least three independent experiments and were analyzed by one-way ANOVA followed by Tukey’s test. (**f** and **g**) Evaluation of the differentiation efficiency of the CDH13-positive iMuSCs in vitro on day 7. (**f**) Representative immunohistochemistry images for MyHC and Hoechst. Scale bars, 200 μm. (**g**) The differentiation efficiency calculated by determining the percentage of Hoechst-positive counts (nuclei) in the MyHC-positive area. Data represent the means + SD of three independent experiments and were analyzed by one-way ANOVA followed by Tukey’s test.

Using the MyoD-hiPSC differentiation system, we also investigated the alternative splicing of *ATP2A1* encoding the SERCA1 protein, which is an intracellular calcium pump located in the SR of skeletal muscle cells^33,34^. However, the splicing defect in the DM1 myotubes was undetectable because the *ATP2A1* exon 22 inclusion was insufficient even in the Cntl myotubes obtained with the MyoD1-induced system (Supplementary Fig. S8a). The low level of *ATP2A1* exon 22 inclusions appeared to be caused by immaturity of the myotubes. We recently reported a transgene-free differentiation culture method to generate MuSCs from hiPSCs^20^, and identified two novel markers for purification of iMuSCs: CDH13 and FGFR4^21^. In particular, CDH13 is expressed in the majority of the myogenic cells and we efficiently acquired iMuSCs from the transgene-free culture. Furthermore, iMuSCs have the potential to generate more mature myotubes than MyoD1-mediated myotubes^22^. Hence, the transgene-free differentiation was conducted using a stepwise protocol to obtain iMuSCs and induce the iMuSCs to differentiate into highly mature myotubes (Fig. 5a). First, two Cntl-hiPSC lines and three DM1-hiPSC lines were cultured for 12–13 weeks according to the previous protocol^20^ (Supplementary Fig. S8b), and the CDH13 expression was analyzed by flow cytometry. CDH13-positive populations in all the cell lines were observed at more than 40% efficiency and were sorted using a cell sorter (Fig. 5b and c). To investigate the properties of the sorted cells, gene expression profiling was performed. RT-qPCR analysis showed that CDH13-positive cells were enriched for *PAX7*, *MYF5*, and *MYOD1* (Fig. 5d). Additionally, the sorted cells showed robust expression of myogenic marker proteins, such as Pax7 and MyoD1 (Fig. 5e and Supplementary Fig. S8c). Finally, re-culture and myogenic differentiation of the sorted cells were conducted, and the CDH13-positive cells from all the cell lines efficiently differentiated into MyHC-positive myotubes (Fig. 5f). The differentiation efficiencies were comparable among these cell lines (Fig. 5g). According to these data, the CDH13-positive iMuSCs were successfully acquired from Cntl and DM1-hiPSCs using the stepwise protocol.

### Myogenic maturation and alternative splicing of SERCA1 in the iMuSC-derived myotubes

**Figure 6:**
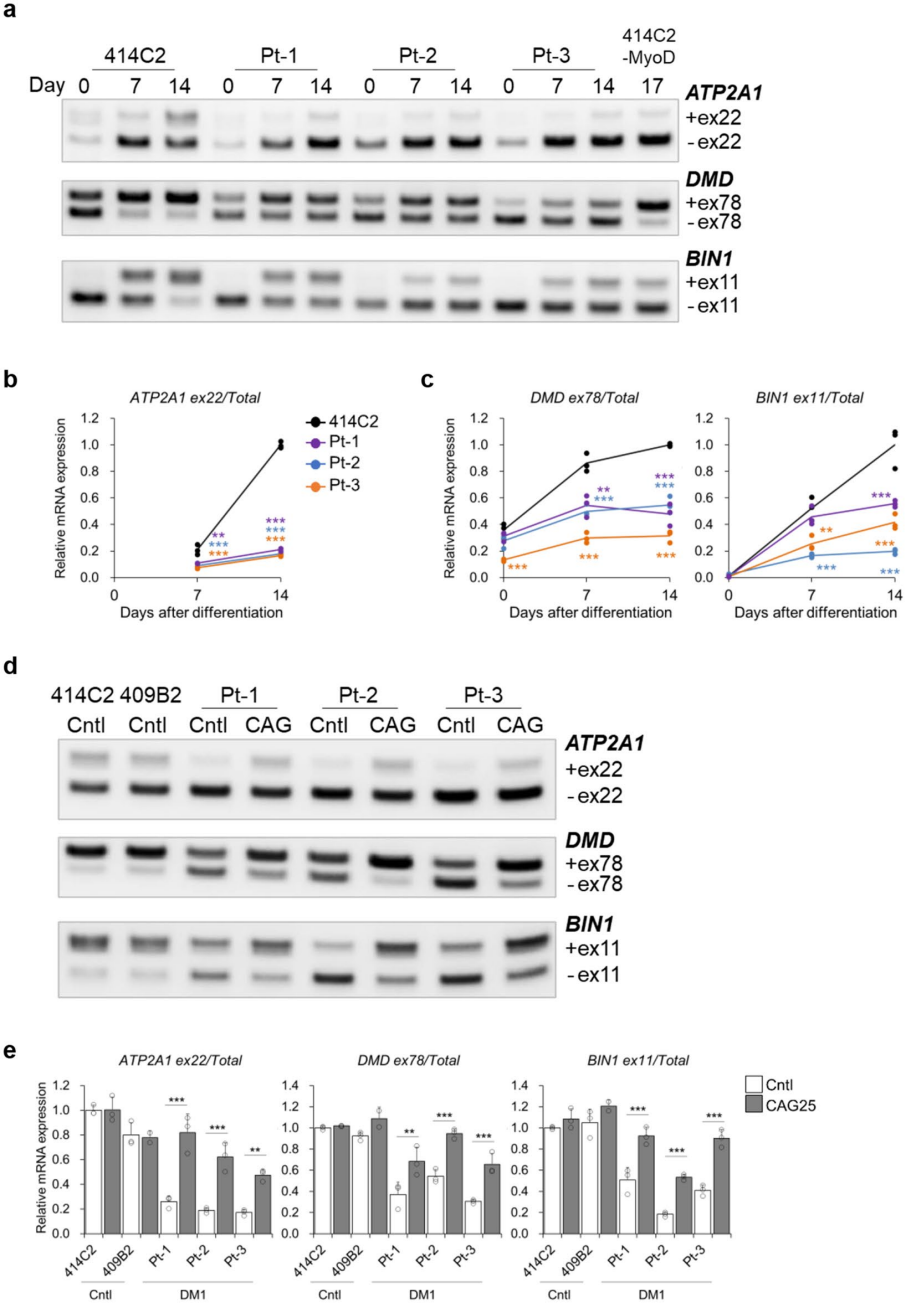
Recapitulation of highly matured myotubes and *ATP2A1* splicing defect in the DM1 cells using iMuSC differentiation system. (**a**–**c**) Evaluation of alternative splicing of *ATP2A1*, *DMD* and *BIN1* on day 0, day 7 and day 14. (**a**) The representative images obtained by gel-based RT-PCR. The target exons are indicated on the right. (**b** and **c**) Results of quantitative analyses of alternative splicing for (**b**) *ATP2A1*, (**c**) *DMD* and *BIN1* by RT-qPCR. The gene expressions are indicated by relative values to 414C2 control on day 14. Data represent the means of three independent experiments and were analyzed by one-way ANOVA followed by Tukey’s test (**P < 0.01, ***P < 0.001, vs. 414C2 control). (**d** and **e**) Evaluation of alternative splicing of *ATP2A1*, *DMD* and *BIN1* after seven days of CAG25 treatment on day 14. (**d**) Representative images obtained by gel-based RT-PCR. (**e**) The results of quantitative analyses by RT-qPCR. The gene expressions are indicated by relative values to 414C2 control treated with control oligonucleotide. Data represent the means + SD of three independent experiments and were analyzed by two-way ANOVA followed by Tukey’s test (**P < 0.01, ***P < 0.001).

To acquire hiPSC-derived myotubes with higher maturity than those obtained using the MyoD1-inducible system, CDH13-positive iMuSCs were induced to differentiate into myotubes in vitro for 14 days. The cells rapidly changed their morphology and exhibited a cylindrical shape by day 7 after differentiation (Supplementary Fig. S9a). Analysis for gene expressions by RT-qPCR showed marked increase in the expressions of *MYH3* and *MYH8* after differentiation (Supplementary Fig. S9b). Interestingly, DM1 myotubes from Pt-2 and Pt-3 showed low expression levels of *MYH8*, whereas they showed strong expression of the fast muscle marker, *MYH1* (Supplementary Fig. S9b). The DM1-related genes *DMPK* and *MBNL1* were expressed in sufficient amounts in the iMuSCs and differentiated myotubes, although expression of *MBNL1* decreased after differentiation (Supplementary Fig. S9c). The *MBNL1* expression levels in the iMuSC-derived myotubes were comparable to those in the MyoD-hiPSC-derived myotubes (Supplementary Fig. S9c). Next, alternative splicing was investigated, and formation of *ATP2A1* exon 22 inclusions in the 414C2 cells was greatly facilitated in a differentiation time-dependent manner (Fig. 6a,b and Supplementary Fig. S9d). Importantly, iMuSC-derived myotubes from the 414C2 on day 14 showed marked elevation of *ATP2A1* exon 22 inclusions in contrast to the case in the MyoD-hiPSC-derived myotubes (Fig. 6a), which enabled clear detection of the splicing defect of the *ATP2A1* transcripts in the DM1 myotubes (Fig. 6a,b and Supplementary Fig. S9d). Accordingly, iMuSC-derived myotubes from the 414C2 cells showed greatly facilitated formation of the *DMD* exon 78 and *BIN1* exon 11 inclusions as compared with the case in the MyoD1-mediated myotubes (Fig. 6a), and their splicing defects were confirmed in the DM1 myotubes (Fig. 6a,c and Supplementary Fig. S9e). In addition, the efficacy of CAG25 against nuclear MBNL1 aggregation and splicing defects was investigated. The results revealed that CAG25 treatment certainly decreased the number of nuclear MBNL1 aggregates from day 5 to day 7 in these DM1 myotubes (Supplementary Fig. S10a–c). The analysis for alternative splicing showed that the Cntl myotubes exhibited a high level of *ATP2A1* exon 22 inclusions as well as *DMD* exon 78 and *BIN1* exon 11 inclusions as compared with the DM1 myotubes (Fig. 6d and e). CAG25 treatment significantly facilitated the formation of *ATP2A1* exon 22, *DMD* exon 78 and *BIN1* exon 11 inclusions in all the DM1 cell lines (Fig. 6d,e and Supplementary Fig. S11a). On the other hand, CAG25 treatment exerted no influence on the expressions of *MYH8*, *DMPK* or *MBNL1* (Supplementary Fig. S11b), so that the efficacy was not caused by a change of their maturity. These data demonstrated that the splicing defect of SERCA1 was successfully recapitulated in vitro using the iMuSC differentiation system.

## Discussion

To understand disease mechanisms and develop therapeutics, human in vitro disease models which properly recapitulate disease-associated phenotypes is essential. In this study, we modified the previously used protocol for MyoD1-induced myogenic differentiation using hiPSCs and also investigated the in vitro differentiation of CDH13-positive iMuSCs for DM1 disease modeling of skeletal muscles. Both myogenic differentiation systems successfully recapitulated two important DM1-related features, nuclear MBNL1 aggregation and alternative splicing defects, in three different DM1 patient-derived myotube populations. Importantly, the simple methods for quantitative analysis to detect the DM1-related phenotypes were established with conventional procedures, namely, immunocytochemistry for nuclear MBNL1 aggregation and RT-qPCR for splicing defects. In the MyoD1-induced system, the quantitative analyses demonstrated that CTGexp-deleted DM1 myotubes generated by CRISPR-Cas9 represented a reversal of these disease phenotypes, comparable to the Cntl myotubes. Furthermore, CAG25 treatment definitely recovered the DM1-related phenotypes in both the MyoD1-induced and iMuSC differentiation systems. These data indicate the reliability of our in vitro hiPSC-based DM1 models.

Previously, we established a replating method with EFS via MyoD1 induction for disease modeling of Duchenne muscular dystrophy^18,35^. In the present modified method, with respect to the previous report^18^, involving direct plating of MyoD-hiPSCs, the doxycycline was transiently removed during differentiation days 4 to 6 to adjust the myogenic differentiation speed and Ara-C was added transiently to remove non-myogenic and proliferative cells. The new modified method not involving replating and EFS led to robust myogenic differentiation on day 17 and clear detection of splicing defects in the DM1-MyoD-hiPSC-derived myotubes. For basic research on DM1, a variety of human in vitro models have been reported over the years, established using DM1 patient-derived cells, e.g., primary dermal fibroblasts^4,36,37^, myoblasts^12,13,38^, urine-derived cells^39^, and hiPSCs^40–42^. Mondragon-Gonzalez et al.^40^ reported an in vitro DM1 model established using patient-derived iPSCs as a tool for drug discovery. In this previous study, the PAX7-GFP conditional expression system by a lentiviral vector was used to induce myogenic differentiation^43^. The system requires purification by selecting GFP-positive cells (PAX7-positive, myogenic progenitors) with a cell sorter after myogenic induction of iPSCs. By contrast, our MyoD1-induced system established using the piggyBac vector enabled us to induce homogeneous and robust myogenic differentiation by a simple culture method without any need for cell sorting with a special equipment. Moreover, unlike our non-viral system, the aforementioned lentiviral system must be managed under P2 level regulation, which can limit easy handling in industrial companies. These differences offer certain advantages to our system, especially for drug screening.

To the best of our knowledge, no simple quantitative method has been established yet for analyzing nuclear MBNL1 aggregation. In this study, we successfully established a simple and reliable quantitation method for nuclear MBNL1 aggregation involving immunocytochemistry after acetone-MeOH fixation, which enabled specific detection of nuclear MBNL1 aggregation in DM1 cells. To evaluate the regulation of endogenous splicing, the vast majority of previous DM1 studies only adopted gel-based RT-PCR, which is considered as a semi-quantitative method. In addition to that method, herein, we also established a quantitative method for analyzing MBNL1-dependent alternative splicing of *DMD*, *BIN1* and *ATP2A1* by RT-qPCR using specific primer sets targeting the mis-spliced exons in the DM1 cells. Importantly, mis-splicing events of *DMD*, *BIN1* and *ATP2A1* are associated with muscle dystrophy, muscle weakness and dysregulation of calcium metabolism, respectively, which are all DM1 manifestations^26,27,34^. In the pathogenesis of DM1, both human studies^7,44^ as well as animal studies^6,45,46^ have identified MBNL1 as the main pathogenic regulator. Therefore, the recovery of endogenous MBNL1 function is necessary for effective DM1 therapies, and our quantitative methods investigating MBNL1-mediated pathology are promising for judging the potential of therapeutics for DM1. Overall, we believe that the combined analyses for nuclear MBNL1 aggregation and splicing defects in the DM1-hiPSCs-derived skeletal myotubes would provide an ideal platform for drug screening against DM1.

SERCA1 is expressed in the SR of skeletal muscles and plays a critical role in the restoration of the calcium pool in the SR and in muscle relaxation after muscle contraction^33^. The protein has two splice variants (adult form SERCA1a: exon 22 + , fetal form SERCA1b: exon 22-) and SERCA1b has almost a half of the Ca^2+^ uptake ability as SERCA1a^47^. Abnormal splicing shift from SERCA1a to SERCA1b is observed in DM1 skeletal muscle cells and the mis-splicing causes intracellular aberrant calcium homeostasis^34^, which can lead to muscle dysfunction. Thus, evaluating SERCA1 alternative splicing in DM1 skeletal muscle cells is meaningful for considering the clinical situation, whereas recapitulation of SERCA1 mis-splicing in vitro seems to require a high level of maturity of the skeletal muscle cells and has been relatively difficult to do, even using primary cell cultures^48,49^. To this end, we investigated the CDH13-positive iMuSCs after in vitro differentiation and the iMuSC-derived myotubes exhibited a more mature state than the MyoD1-mediated myotubes, which enabled us to clearly recapitulate the SERCA1 splicing defect in the DM1-iMuSC-derived myotubes. Dastidar et al.^50^ reported observing SERCA1 splicing defect in DM1-hiPSC-derived myotubes established via mesoangioblast-like cell induction in a six week culture, and thus long-term culture seems to be required for recapitulation of the SERCA1 splicing defect. We confirmed that cryopreserved CDH13-positive iMuSCs stably differentiated into myotubes on 96-well microplates; hence, the system is available for selection of candidate drugs for DM1 therapy. Interestingly, the myotubes derived from Pt-2 and Pt-3 showed low expression levels of *MYH8*, but high expression levels of *MYH1* as compared with the Cntl myotubes. A perturbed expression profile of MyHC genes was reported by microarray analysis of skeletal muscle biopsies from DM1 patients^51^, and the iMuSC-derived myotubes from the patients may exhibit an intriguing phenotype in the skeletal muscles of DM1 patients.

DM1 is a multisystemic disorder mainly characterized by dysfunction of skeletal muscles, heart, and brain, and we investigated the disease modeling of skeletal muscles focusing on MBNL1-related molecular processes. Meanwhile, previous reports show that abnormal activation of CELF1 is seen in DM1 heart tissues^52^ and deregulation of CELF1 is involved in aberrant cardiac conduction^53,54^. Moreover, the mice model expressing 960 CTG repeats in the *DMPK* gene in skeletal muscles is reported to overexpress CELF1 and manifest muscle wasting^55^. To develop DM1 therapies, individual disease modeling of these affected tissues in DM1 is necessary, and the molecular analyses of CELF1-related pathways would be required in addition to those of MBNL1-related pathways. In regard to tissue-specific disease modeling, the patient-derived iPSC technology offers the powerful advantages of pluripotency. Differentiation protocols for cardiomyocytes^56^ and neuronal cells^57^ have been established, and the iPSC platforms are valuable for a more comprehensive understanding of DM1. Patient-derived cells are expected to study the wide phenotype spectrum of DM1 patients, however, the length of CTG repeats in all DM1-hiPSCs used in this study was relatively long (around and over 1,000 repeats) and all the hiPSCs were derived from female patients. To overcome this limitation, further studies using other DM1-hiPSC lines with various backgrounds would be needed to deepen understanding the pathogenetic mechanism of DM1.

In conclusion, we successfully established in vitro skeletal muscle models of DM1 from patient-derived iPSCs using the MyoD1-induced system and iMuSC differentiation system. These induction systems allowed the crucial DM1-related phenotypes of nuclear MBNL1 aggregation and alternative splicing to be recapitulated, as validated by well-organized quantitative methods. The MyoD1-induced system established in a short-term culture is promising for DM1 drug screening, and the iMuSC differentiation system, which led to the formation of myotubes with a higher maturity and enabled the detection of SERCA1 splicing defect, which is expected to be useful to predict the clinical efficacies of candidate drugs.

## Methods

### Ethical approval

This study was conducted with the approval of the Ethics Committee of the Graduate School of Medicine, Kyoto University, Kyoto University Hospital (The approved number R91), and Taisho Pharmaceutical Co., Ltd (The approved number 19-12, 20-10, 21-06 and 22-07). Written informed consent was obtained from the patients according to the principles laid down in the Declaration of Helsinki. All procedures were performed according to the relevant guidelines and regulations approved by the Institutional Review Board at Kyoto University.

### Human iPSC lines and maintenance culture

The previously established Cntl-hiPSC lines, 414C2 and 409B2^58^ (healthy donor), and three DM1-hiPSC lines^19^ (DM1 patients) were used for all the experiments. The information of these DM1 patients is listed in Supplementary Table S1. All the hiPSC lines were generated using episomal vectors and maintained under feeder-free culture conditions, as described in previous reports^59,60^. Briefly, the cells were plated onto an iMatrix-511 (Nippi, Tokyo, Japan) pre-coated 6-well plate (1 × 10^4^ cells/well) and cultured in StemFit AK02N medium (Ajinomoto, Tokyo, Japan). The cells were passaged once a week using Accutase (Nacalai Tesque, Kyoto, Japan).

### Myogenic differentiation from MyoD1-induced hiPSC lines (MyoD-hiPSC)

The generation of hiPSC lines expressing Tet-inducible MyoD1 was performed as described in a previous report^16,19^. After transfection of MyoD1-expressing piggyBac vector, puromycin (Nacalai) or G418 (Nacalai) -resistant clones were used for the subsequent experiments. The MyoD1-induced hiPSC lines (MyoD-hiPSCs) were dissociated with Accutase and plated on to 1% Matrigel (Corning, New York, United States)-coated multi-well plates or an 8-well μ-slide (ibidi, Gräfelfing, Germany) in StemFit + 10 μM Y-27632 (Wako, Osaka, Japan) (2.5–3.5 × 10^4^ cells/cm^2^). After 24 and 48 h, the media were replaced with Primate ES Cell Medium (PECM, Reprocell, Kanagawa, Japan) and PECM + 0.1–1.0 μg/mL Doxycycline (Dox) (LKT Laboratories, Minnesota, United States), respectively. The next day, the media were replaced with 5% knockout serum replacement (KSR, Thermo Fisher Scientific, Massachusetts, United States) in αMEM (Nacalai) + Dox + 200 μM 2-mercaptoethanol (2-ME, Nacalai). On differentiation day 4, the medium was replaced with 2% horse serum (HS, Sigma-Aldrich, Massachusetts, United States) in αMEM + 200 μM 2-ME, followed by incubation for two days. Thereafter, the medium was replaced with 2%HS/αMEM containing Dox, 200 μM 2-ME, 10 ng/mL recombinant human IGF-1 (PeproTech, New Jersey, United States), and 5 μM SB431542 (SB, Sigma-Aldrich) and changed every other day. After four days of culture, the medium was replaced with 2%HS/αMEM or DMEM-High glucose (Nacalai) + 200 μM 2-ME + 10 ng/mL IGF-1 + 5 μM SB containing 1 μM Ara-C (Sigma-Aldrich), or not. After three days of the culture, the media were replaced with 2%HS/DMEM + 200 μM 2-ME + 10 ng/mL IGF-1 + 5 μM SB and changed every other day until day 17 of differentiation. Cultured cells were visualized and photographed under an IX73 Inverted Microscope (Olympus, Tokyo, Japan).

### Generation of the CTGexp-deleted hiPSC line

First, to check the cleavage activity at the 5' (sgRNA1 target site: gctcgaagggtccttgtagcCGG) and 3' (sgRNA2 target site: gctgaggccctgacgtggatGGG) regions of the CTG repeat on the *DMPK* gene, the sgRNA-expressing plasmid vectors (pHL-H1-DMPK-3'UTR-gRNA1-mEF1a-RiH, and pHL-H1-DMPK-3'UTR-gRNA2-mEF1a-RiH) were constructed by inserting the each sgRNA into the BamHI and EcoRI site of the pHL-H1-ccdB-mEF1a-RiH vector (Addgene plasmid # 60601), as described in a previous report^61^. Next, sgRNA was prepared by in vitro transcription (IVT) reaction based on our previous reports^62,63^. In brief, template DNA was prepared by PCR reaction using the T7- DMPK-3’UTR-gRNA1-fwd and sgRNA- + 85 rev primers for gRNA1, and the T7- DMPK-3’UTR-gRNA2-fwd and sgRNA- + 85 rev primers for gRNA2. The PCR product was gel-extracted and purified using Wizard SV Gel and PCR Clean-Up System (Promega, Wisconsin, United States). 150 nM of the template PCR product was used for the IVT reaction using the MEGAshortscript T7 Transcription kit (Thermo Fisher Scientific) with 75 mM each T7 NTPs and T7 Enzyme Mix (incubated at 37 °C for 12–18 h). After the TURBO DNase treatment (37 °C for 15 min), the gRNAs were purified with the RNeasy MinElute Cleanup Kit (QIAGEN, Hilden, Germany) and utilized for the electroporation experiments.

For the electroporation of the Cas9 ribonucleoprotein and gRNA complex, 10 µg of Cas9 protein (Alt-R® S.p. Cas9 Nuclease, Integrated DNA Technologies, Iowa, United States) was mixed with the 1.25 µg of IVT gRNA1 and 1.25 µg of gRNA2 in advance, and MaxCyte electroporation was performed into 2.5 × 10^6^ cells per 50 μL of the MyoD-hiPSCs (CiRA00112) with the Optimization Energy 8 protocol using the OC-100 Processing Assembly. After expansion of the electroporated bulk MyoD-CiRA00112, genomic DNAs were extracted and PCR amplification was performed using the DMPK-ex15-3’UTR-fwd2 and DMPK-ex15-3’UTR-rev2 primers to assess the deletion efficiency by gel electrophoresis.

T7- DMPK-3’UTR-gRNA1-fwd

GAAATTAATACGACTCACTATAGgctcgaagggtccttgtagcGTTTTAGAGCTAGAAATAGCAAG

T7- DMPK-3’UTR-gRNA2-fwd:

GAAATTAATACGACTCACTATAGgctgaggccctgacgtggatGTTTTAGAGCTAGAAATAGCAAG

sgRNA- + 85 rev:

AAAGCACCGACTCGGTGCCACTTTTTCAAGTTGATAACGGACTAGCCTTATTTTAACTTGCTATTTCTAGCTCTAAAAC

DMPK-ex15-3’UTR-fwd2

ACCCTAGAACTGTCTTCGACTCC

DMPK-ex15-3’UTR-rev2

TTCCCGAGTAAGCAGGCAGAG

After confirming the bulk deletion efficiency in MyoD-CiRA00112, 34 subclones were isolated. Genomic DNA was extracted from each clone for genotyping with a GenElute™ Mammalian Genomic DNA Miniprep Kit (Sigma-Aldrich), in accordance with the manufacturer’s protocol. Genomic PCR was performed using the DMPK-ex15-3’UTR-fwd2 and DMPK-ex15-3’UTR-rev2 primers to identify a CTGexp-deleted cell line. The genomic PCR was performed with initial denaturation at 94 °C for one minute and 30 cycles of 94 °C for 30 s, 62 °C for 30 s and 72 °C for 3 min, followed by 72 °C for 5 min, with a TaKaRa LA Taq (Takara Bio, Shiga, Japan) and a thermal cycler (Applied Biosystems, California, United States). The PCR products (20 ng/sample) were stained with GelRed (Biotium, California, United States) and applied on a 0.8% agarose gel (Nacalai). The scanned images were obtained with ChemiDoc (BIO-RAD, California, United States). Thereafter, the gels containing the target bands were excised out and the DNA was extracted from the gels with the Wizard™ SV Gel and PCR Clean-Up System (Promega), in accordance with the manufacturer’s protocol. The sequences of these clones were confirmed with the primer sets, and one clone was selected for the subsequent experiments.

### Treatment of oligonucleotides

Two morpholino oligonucleotides were purchased from Gene Tools (Oregon, United States). The sequence of CAG25^32^ was 5′-AGCAGCAGCAGCAGCAGCAGCAGCA-3′, and that of the inverted control was 5′-ACGACGACGACGACGACGACGACGA-3′. The cells were treated with CAG25 (3–30 μM) or inverted control oligonucleotide (30 μM) together with the transfection reagent Endo-Porter (6 μM, Gene Tools), in accordance with the manufacturer’s instructions. Cells were analyzed two days after treatment by the nuclear MBNL1 aggregation assay, or after 4–7 days by the splicing assay.

### Myogenic differentiation by the stepwise protocol

For myogenic induction of hiPSCs to obtain iMuSCs, we followed our previously reported protocol^20^. Undifferentiated hiPSCs were plated on to a Matrigel-coated 6-well plate in StemFit + 10 μM Y-27632 (1 × 10^4^ cells/well). After two days, the medium was replaced with fresh Stemfit and cultured for one day. The medium was replaced with CDMi + 10 μM CHIR99021 (CHIR, Wako) + 5 μM SB. CDMi is composed of IMDM (Wako) and Ham's F-12 (Wako) at the ratio of 1:1 supplemented with 1% bovine serum albumin (BSA) (Sigma-Aldrich), 1% Penicillin Streptomycin Mixed Solution (Nacalai), 1% CD Lipid Concentrate (Thermo Fisher Scientific), 1% Insulin-Transferrin Selenium (Thermo Fisher Scientific), and 450 μM 1-Thioglycerol (Sigma-Aldrich). After seven days of differentiation, the cells were dissociated with Accutase and plated on to a Matrigel-coated 6-well plate (4.5 × 10^5^ cells/well) in CDMi medium + 10 μM CHIR + 5 μM SB + 10 μM Y-27632. On differentiation day 14, the cells were dissociated with Accutase again and placed onto a Matrigel-coated 6 well plate (8 × 10^5^ cells/well) in CDMi medium + 10 μM Y-27632. After three days, the medium was replaced with serum-free culture medium (SF-O3; Sanko Junyaku, Tokyo, Japan) supplemented with 0.2% BSA, 200 μM 2-ME, 10 ng/mL IGF-1, 10 ng/mL recombinant human bFGF (Oriental Yeast, Tokyo, Japan) and 10 ng/mL recombinant human HGF (PeproTech). The medium was changed twice a week until day 35 of the differentiation. On differentiation day 35, the medium was replaced with 2%HS/DMEM supplemented with 200 μM 2-ME, 10 ng/mL IGF-1, 5 μM SB, 0.5% Penicillin Streptomycin Mixed Solution, and 2 mM L-glutamine (Nacalai). Thereafter, the medium was changed every 2–3 days and the cells were cultured in the medium until week 12–13 of differentiation.

### Immunocytochemistry

The cells were fixed with 4% PFA in PBS (Nacalai) at RT for 10 min and permeabilized with 1% Triton (Nacalai) in PBS at RT for 30 min. Then, the cells were washed twice with 0.1% Tween-20 (Sigma- Aldrich) in PBS (PBS-T) and blocked with blocking buffer (3% BSA in PBS-T) at RT for one hour. Thereafter, the cells were incubated with the appropriate primary antibodies diluted in blocking buffer overnight at 4 °C. The following day, the cells were washed three times with PBS-T for 10 min and incubated with the appropriate secondary antibodies diluted in blocking buffer for one hour. After incubation with PBS-T containing nuclear counterstain (Hoechst 33,342, 1:5000, Thermo Fisher Scientific) at RT for 10 min, the cells were washed four times with PBS-T and once with PBS for 10 min. To perform quantitative analysis for nuclear MBNL1 aggregation, the cells were fixed and permeabilized with pre-chilled 50% acetone (Nacalai)/50% MeOH (Nacalai) at 4 °C for 5 min. Then, the cells were washed twice with PBS and blocked with blocking buffer at RT for one hour. Thereafter, antibody reaction was performed as described above. All antibodies used in this study are listed in Supplementary Table S2. The samples were visualized and photographed under a BZ-X700 (Keyence, Osaka, Japan) or LSM700 confocal microscope (Carl Zeiss, Oberkochen, Germany). The images of the immunostained samples taken under the BZ-X700 (× 10 objective) were analyzed using the BZ-X analyzer software (Keyence, Osaka, Japan) to measure the MyHC-positive area. The differentiation efficiency was determined by calculating the percentage of Hoechst-positive counts (nuclei) in the MyHC-positive area. Three images were taken from each well and analyzed.

### Quantitative analysis for nuclear MBNL1 aggregation

For analysis to quantify nuclear MBNL1 aggregation, the images of the immunostained samples after acetone-MeOH fixation taken under the LSM700 confocal microscope (× 40 oil immersion objective) were analyzed using the ImageJ software (National Institutes of Health, Maryland, United States). The spot-like signals of MBNL1 within the nuclear regions were counted using the Find Maxima process. The nuclear regions were detected by Hoechst counterstaining. The number of MBNL1 spot-like signals per nucleus and the percentage of nuclei with more than three MBNL1 spot-like signals were calculated using the analyzed data. Five images were obtained from each well and analyzed.

### FISH

After fixing with 4% PFA in PBS, the cells were permeabilized with 0.3% Triton in PBS at RT for 10 min. The cells were washed twice with 5 mM MgCl2 (Sigma-Aldrich) in PBS and incubated with in situ hybridization buffer at 37 °C for 90 min. The in situ hybridization buffer is composed of 100 nM Cy3-labeled 2’OMe (CAG)7 (Gene Design, Osaka, Japan), 2 × SSC (Nacalai), 10% dextran sulfate (Merck, Darmstadt, Germany), 40% formamide (Sigma-Aldrich), 0.2% BSA, 0.1 mg/ml herring sperm DNA (Promega), 0.1 mg/ml baker's yeast transfer RNA (Sigma-Aldrich) and 4 mM ribonucleoside vanadyl complexes (Sigma-Aldrich). Then, the cells were washed with PBS-T at RT for 5 min and at 45 °C for 30 min. Thereafter, immunocytochemistry for MBNL1 and nuclear counterstain were performed as described above, except that anti-MBNL1 antibody was incubated at RT for one hour. After fixing with acetone-MeOH, immunocytochemistry for MBNL1 was performed using antibodies diluted in 2 × SSC, as described in a previous report^25^. The labeled cells were treated with 4% PFA + 5 mM MgCl2 in PBS at RT for one minute and 40% formamide in 2 × SSC for one minute. Then, in situ hybridization and nuclear counterstain were performed as described above. The samples were visualized and photographed under a BZ-X700 (× 100 objective).

### Gel-based RT-PCR

Total RNA was extracted with a ReliaPrep RNA Cell Miniprep system (Promega) and cDNA synthesis was performed with a ReverTra Ace qPCR RT kit (TOYOBO), in accordance with the manufacturer’s protocol. RT-PCR for alternative splicing analysis of *DMD*, *BIN1*, and *ATP2A1* was performed with initial denaturation at 94 °C for 5 min and 35 cycles of 94 °C for 30 s, 60 °C for 30 s and 72 °C for one minute, followed by 72 °C for 5 min, with a TaKaRa Ex Taq (Takara Bio) and the thermal cycler (Applied Biosystems), as previously described^19^. PCR products (5 μg/sample) were stained with GelRed and applied on a 2% agarose gel (Nacalai). The scanned images were obtained with ChemiDoc (BIO-RAD). All primer sets used in this study are listed in Supplementary Table S3.

### Quantitative Real-time RT-PCR (RT-qPCR)

Quantitative Real-time RT-PCR (RT-qPCR) was performed from 4 ng of cDNA per sample with a SYBR Green system (Applied Biosystems) and a StepOnePlus (Applied Biosystems) or a QuantStudio 3 (Applied Biosystems), in accordance with the manufacturer’s protocol. The assay was performed in duplicate for each sample. *CSNK2A2* was used as the internal control^64^. For quantitative analysis of alternative splicing for *DMD*, *BIN1*, and *ATP2A1*, the target gene expression with a specific exon was normalized to the total expression level of each target gene. The primer sets used in this study are listed in Supplementary Table S3.

### Sample preparation for flow cytometry

The cells after 12–13 weeks of differentiation by the stepwise protocol were washed with PBS and treated with Collagenase mix solution at 37℃ for 7 min. Collagenase mix solution is composed of DMEM supplemented with 0.05% Collagenase H (Meiji Seika Pharma, Tokyo, Japan) and 0.5% Collagenase G (Meiji Seika Pharma). Accutase was added to the solution and incubated at 37 °C for 10 min. The solution was neutralized with DMEM + 10% HS and centrifuged at 380 × *g*, 4 °C for 7 min. After removal of the supernatant, the cells were resuspended in DMEM + 10% HS and filtered through a 40-µm cell strainer (Corning). To remove debris and dead cells, density gradient centrifugation was performed using Optiprep (Serumwerk Bernburg, Bernburg, Germany) in DMEM + 10% HS (ρ = 1.11 g/mL), in accordance with the manufacturer’s protocol. The fraction of living cells was resuspended in HBSS + 1% BSA and cytometry was performed. The cells were incubated with an APC-conjugated anti-CDH13 antibody (5 × 10^6^ cells/mL) on ice for 15 min. Thereafter, they were diluted in HBSS + 1% BSA and centrifuged at 380 × *g*, 4 °C for 5 min. After removal of the supernatant, the cells were resuspended in HBSS + 1% BSA containing Hoechst 33342 (1:2000, 5 × 10^6^ cells/mL) and filtered with a Round-Bottom Tube with Cell Strainer (Corning). The prepared cells were stocked on ice before the flow-cytometric analysis.

### Flow-cytometric analysis and sorting

Flow-cytometric analysis was performed using Aria II (BD, New Jersey, United States), in accordance with the manufacturer’s protocol. Gating of the CDH-13-positive fraction was determined using unstained cells as the baseline control. After sorting of the CDH-13-positive cells in DMEM + 2% HS + 10 μM Y-27632, the suspension was centrifuged at 780 × *g*, 4 °C for 10 min. The supernatant was removed and the cells were resuspended in DMEM + 2% HS and cytometry was performed. To investigate the properties of the sorted cells, the cells were either processed for RT-qPCR or immunocytochemistry. In immunocytochemistry, the images of the immunostained samples taken under the BZ-X700 (× 20 objective) microscope were analyzed using a BZ-X analyzer software to calculate the percentage of Pax7- and MyoD1-positive cells. For cryopreservation, the 2 × 10^5^ sorted cells were resuspended in 200 μL of Bambanker (NIPPON Genetics, Tokyo, Japan), and were transferred into 1.0 mL storage frozen vial (Thermo Fisher Scientific). Then, the cells were cooled to − 80 °C at − 1 °C/min by a program freezer (VIA Freeze, Cytiva, Massachusetts, United States) and placed in liquid nitrogen for extended periods of time.

### Re-culture and myogenic differentiation of the sorted cells

Frozen sorted cells were thawed rapidly in a water bath at 37 °C. The cells were suspended in in StemFit + 10 μM Y-27632 and centrifuged at 780 × *g*, RT for 5 min. After removal of the supernatant, the cells were resuspended in StemFit + 10 μM Y-27632 and plated on to a 1% Matrigel-coated 96-well plate (5 × 10^3^ cells/well). After two days, the medium was replaced with StemFit and changed every other day. When the cells became fully confluent, the medium was replaced with DMEM-High glucose/pyruvate (Thermo Fisher Scientific) supplemented with 10 ng/mL IGF-1, 5 μM SB and 1% N-2 Supplement (Thermo Fisher Scientific). Thereafter, the medium was changed every 3–4 days and the cells were cultured in the medium until day 7 or 14 of differentiation. Cultured cells were visualized and photographed under the IX73 Inverted Microscope (Olympus).

### Statistics

For all experiments, at least three independent experiments were performed in duplicate. All data were included with individual data plots and indicated as the means + SD, except for the time-course data, which are expressed as the means. Between-group comparisons were performed by the paired t-test or ANOVA, followed by Tukey’s multiple comparisons using the EXSUS software (CAC Croit, Tokyo, Japan). *P* < 0.05 was considered as denoting significance.

## Supplementary Information

Supplementary Information.

## Data Availability

The data will be made available by the corresponding author upon reasonable request.
