# Supplementary material for: Establishment of quantitative and consistent in vitro skeletal muscle pathological models of myotonic dystrophy type 1 using patient-derived iPSCs
Source: Sci Rep. 2023 Jan 11;13:94. doi: 10.1038/s41598-022-26614-z (PMC9834395; doi:10.1038/s41598-022-26614-z)
Supplement: Supplementary file 1 — Supplementary Information. [file 41598_2022_26614_MOESM1_ESM.pdf]

Supplementary Information

Establishment of quantitative and consistent in vitro skeletal muscle pathological models of myotonic dystrophy type 1 using patient-derived iPSCs

Authors

Ryu Kawada[1,2], Tatsuya Jonouchi[1], Akihiro Kagita[1], Masae Sato[1], Akitsu Hotta[1], Hidetoshi Sakurai[1]*

*Correspondence: hsakurai@cira.kyoto-u.ac.jp

Affiliation

[1] Department of Clinical Application, Center for iPS Cell Research and Application (CiRA), Kyoto University, Kyoto 606-8507, Japan

[2] Discovery Research Laboratories, Taisho Pharmaceutical Co., Ltd., Saitama, 331-9530, Japan.

Supplementary Figures

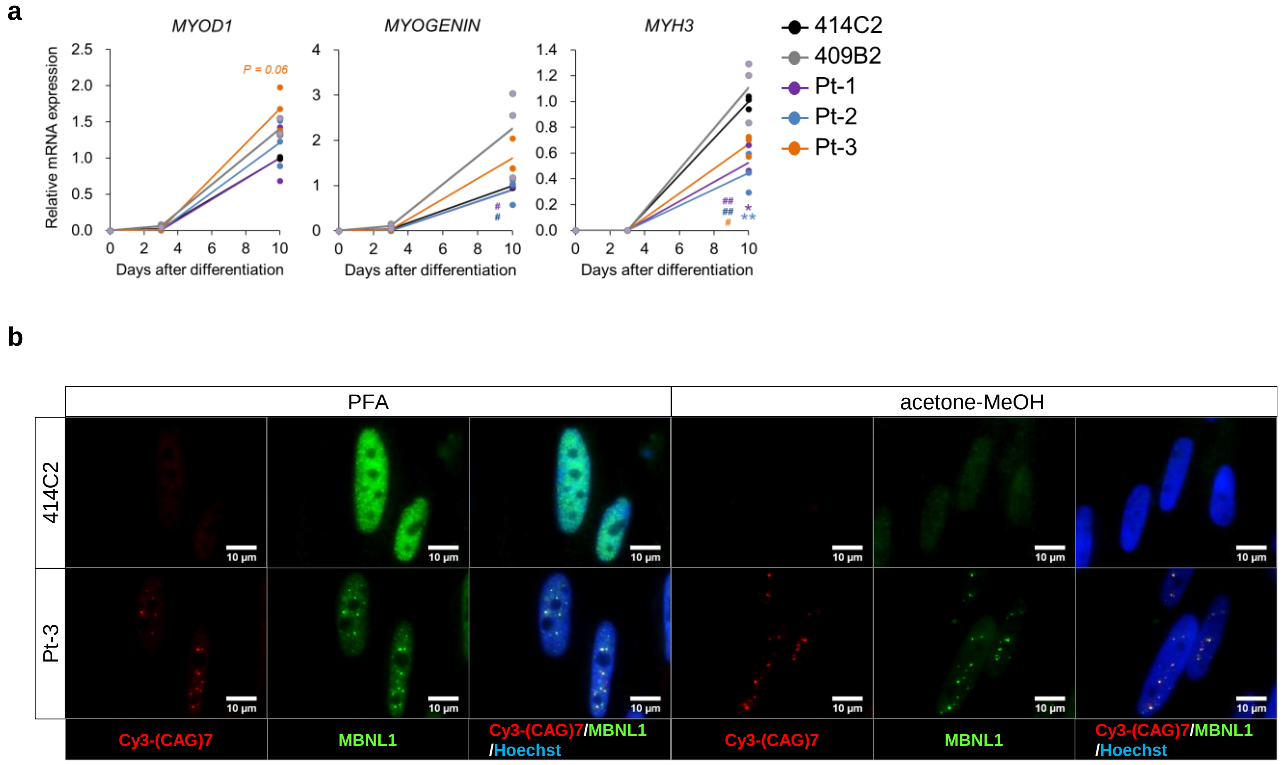

Supplementary Figure S1. Colocalization of nuclear MBNL1 aggregation and CUGexp foci in the myotubes differentiated from MyoD-DM1-hiPSC. Related to Figure 1

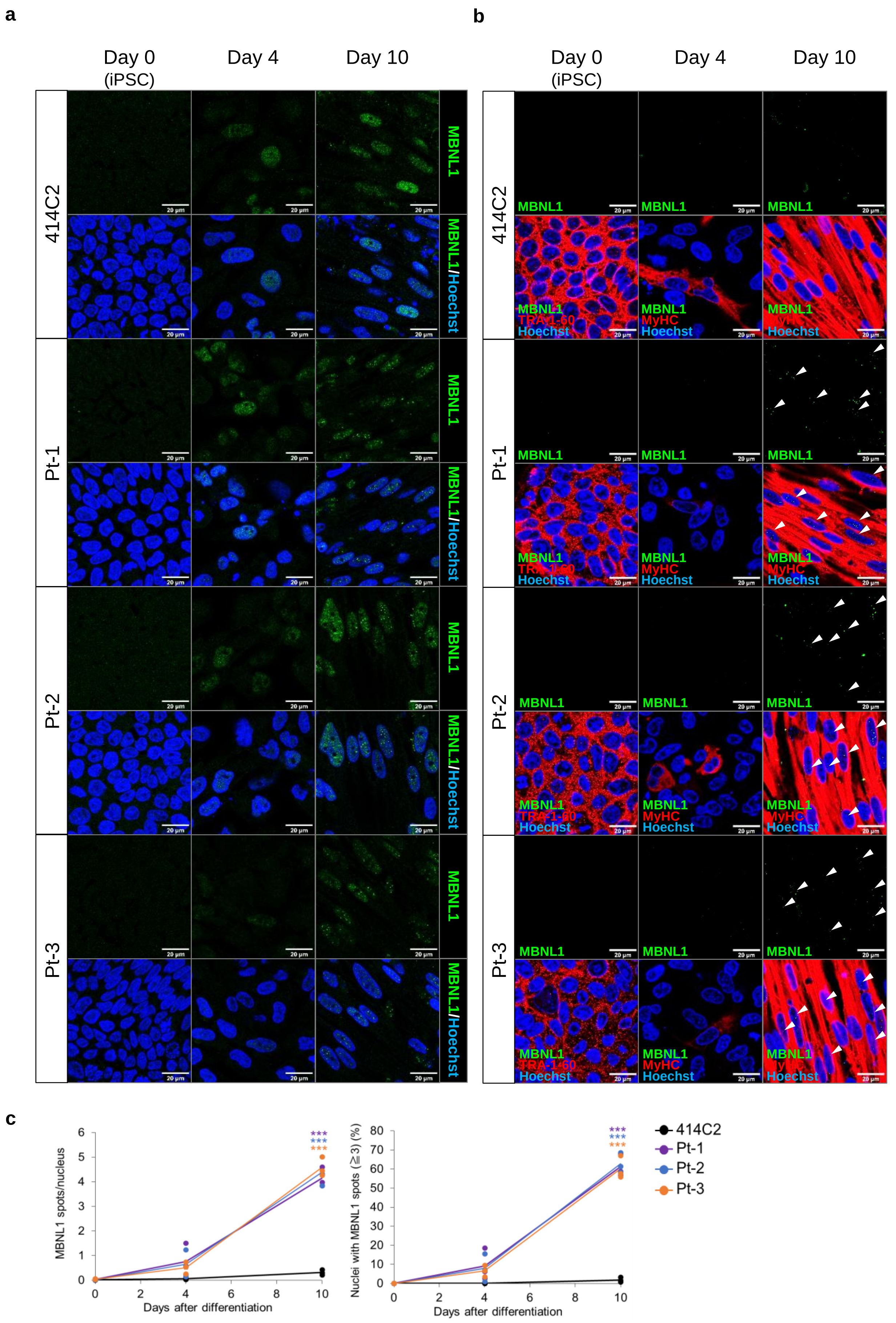

Supplementary Figure S2. Time-course analysis of nuclear MBNL1 aggregation in MyoD-hiPSCs. Related to Figure 1

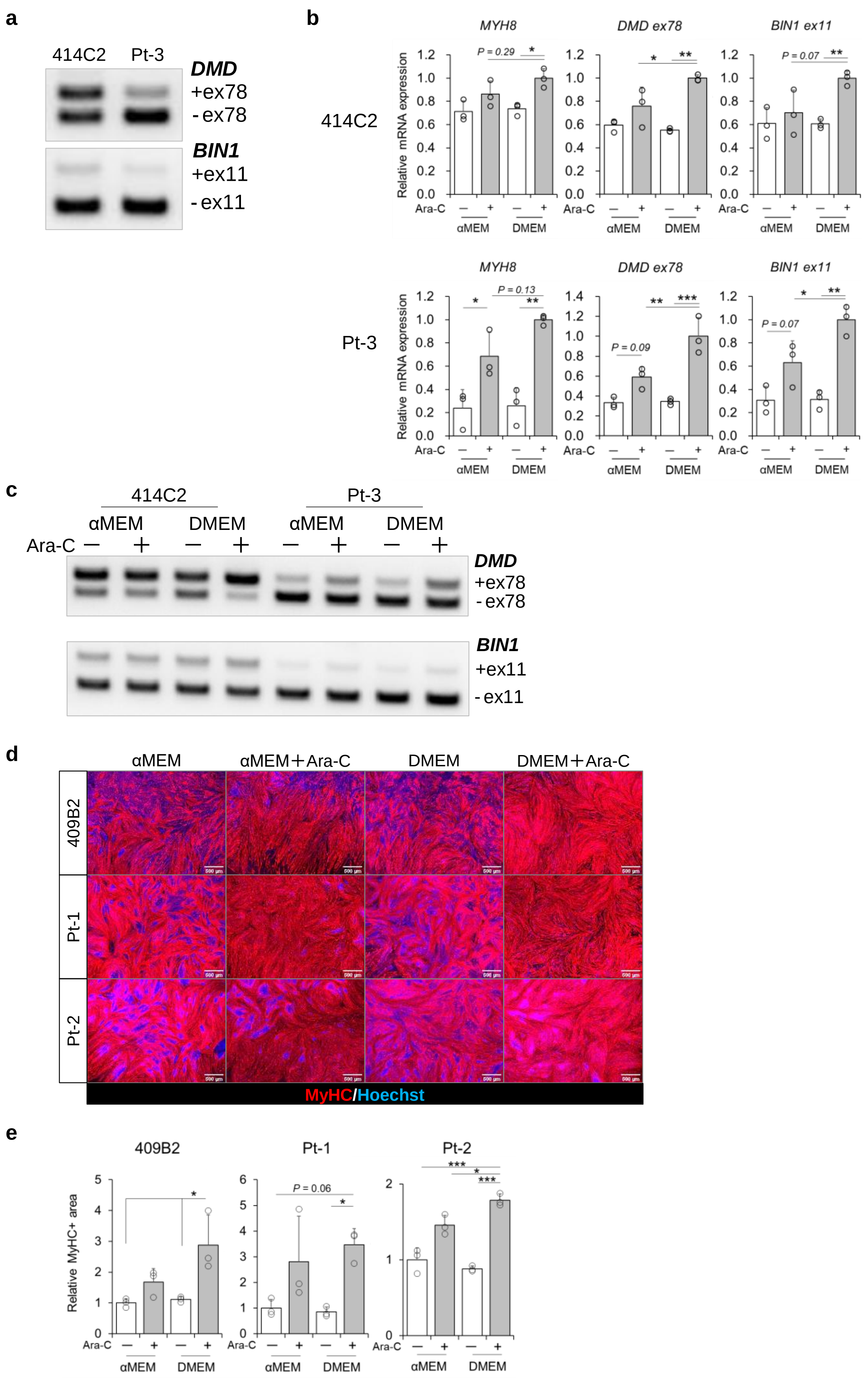

Supplementary Figure S3. Modification of the MyoD1-induced system to recapitulate the splicing defects in the DM1 cells. Related to Figure 2

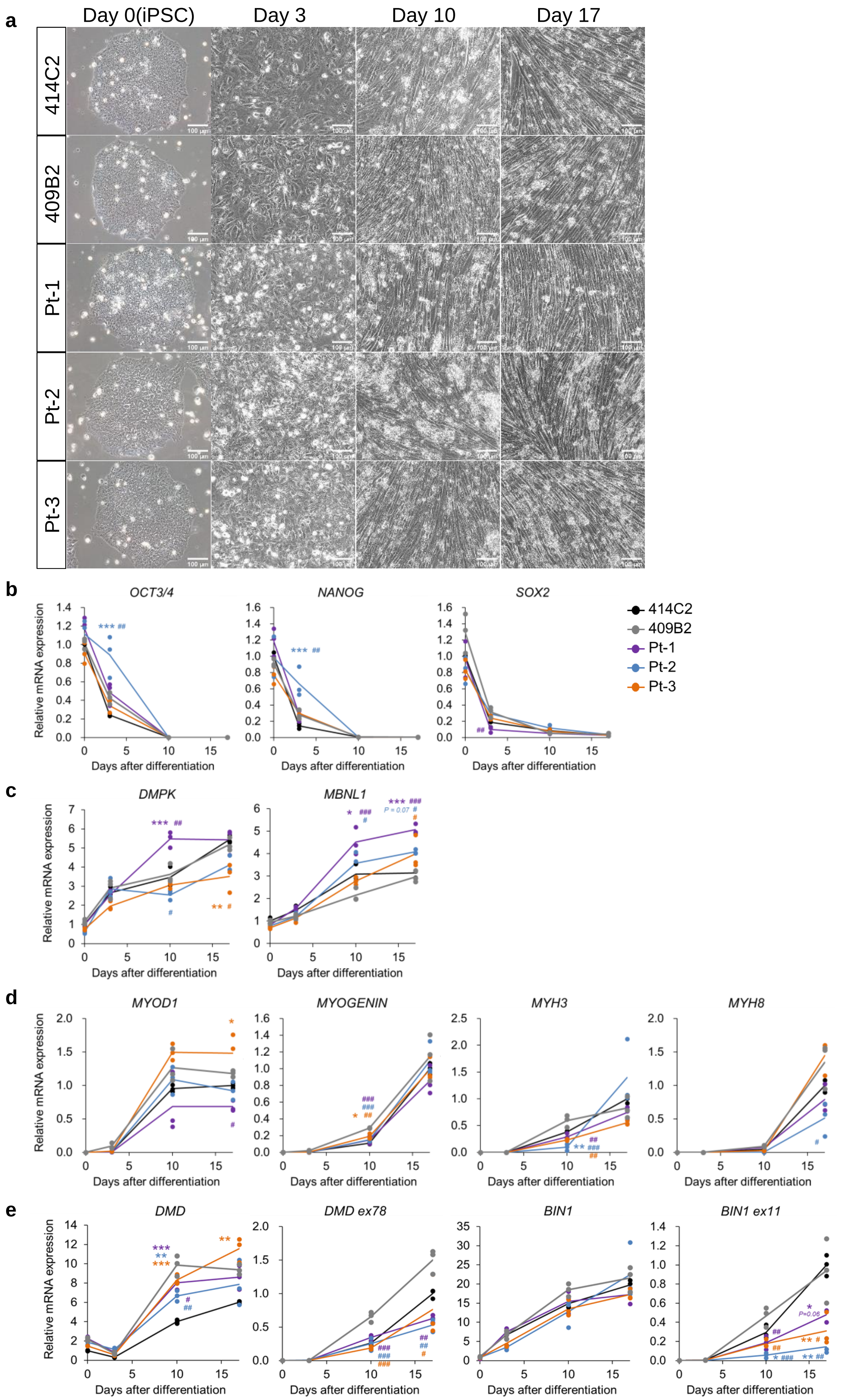

Supplementary Figure S4. Time-course analysis of gene expressions with the use of the newly modified protocol for MyoD1-induced system. Related to Figure 2

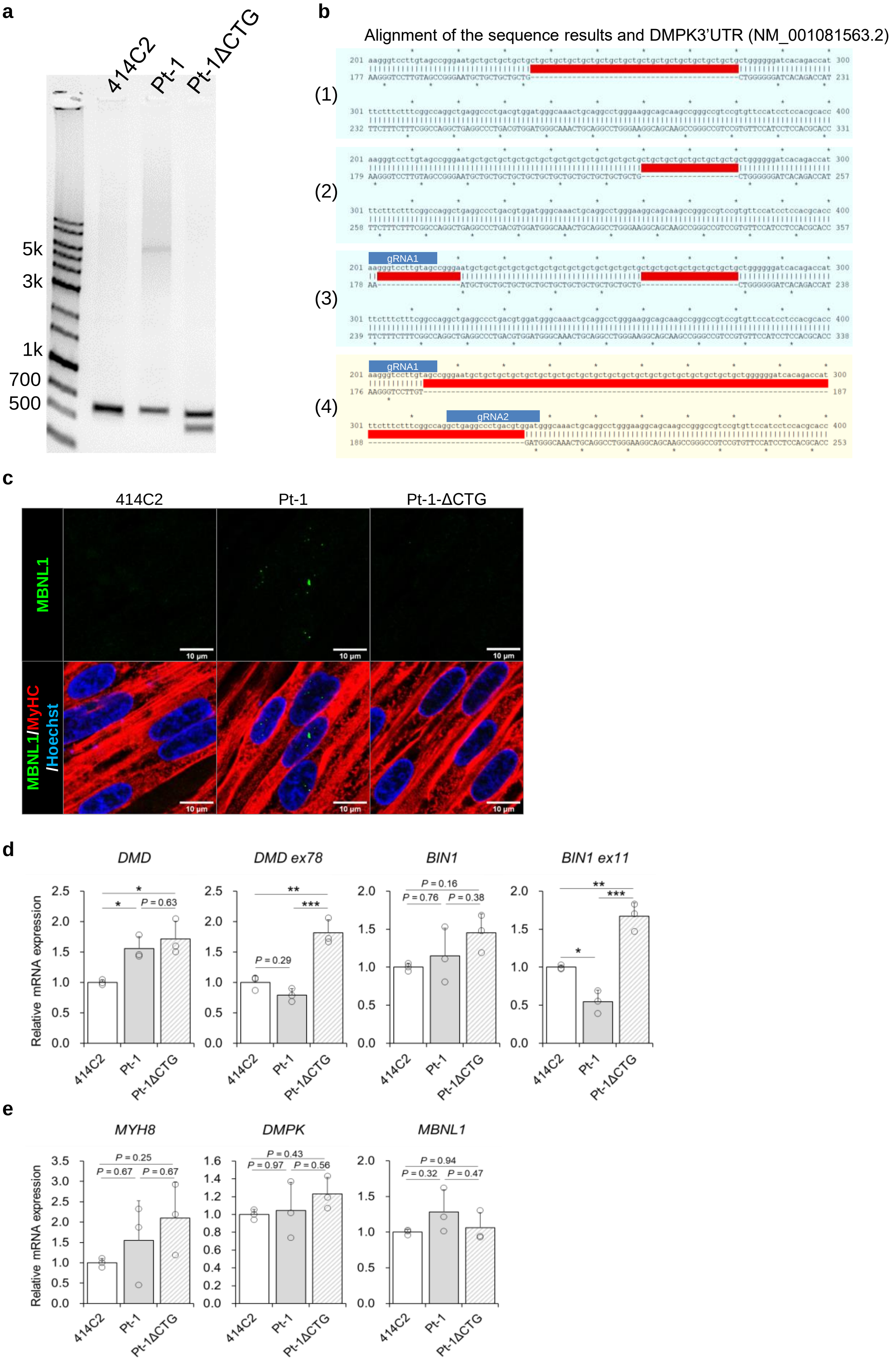

Supplementary Figure S5. Analyses of DM1 phenotypes using CTGexp-deleted MyoD-DM1-hiPSCs. Related to Figure 3

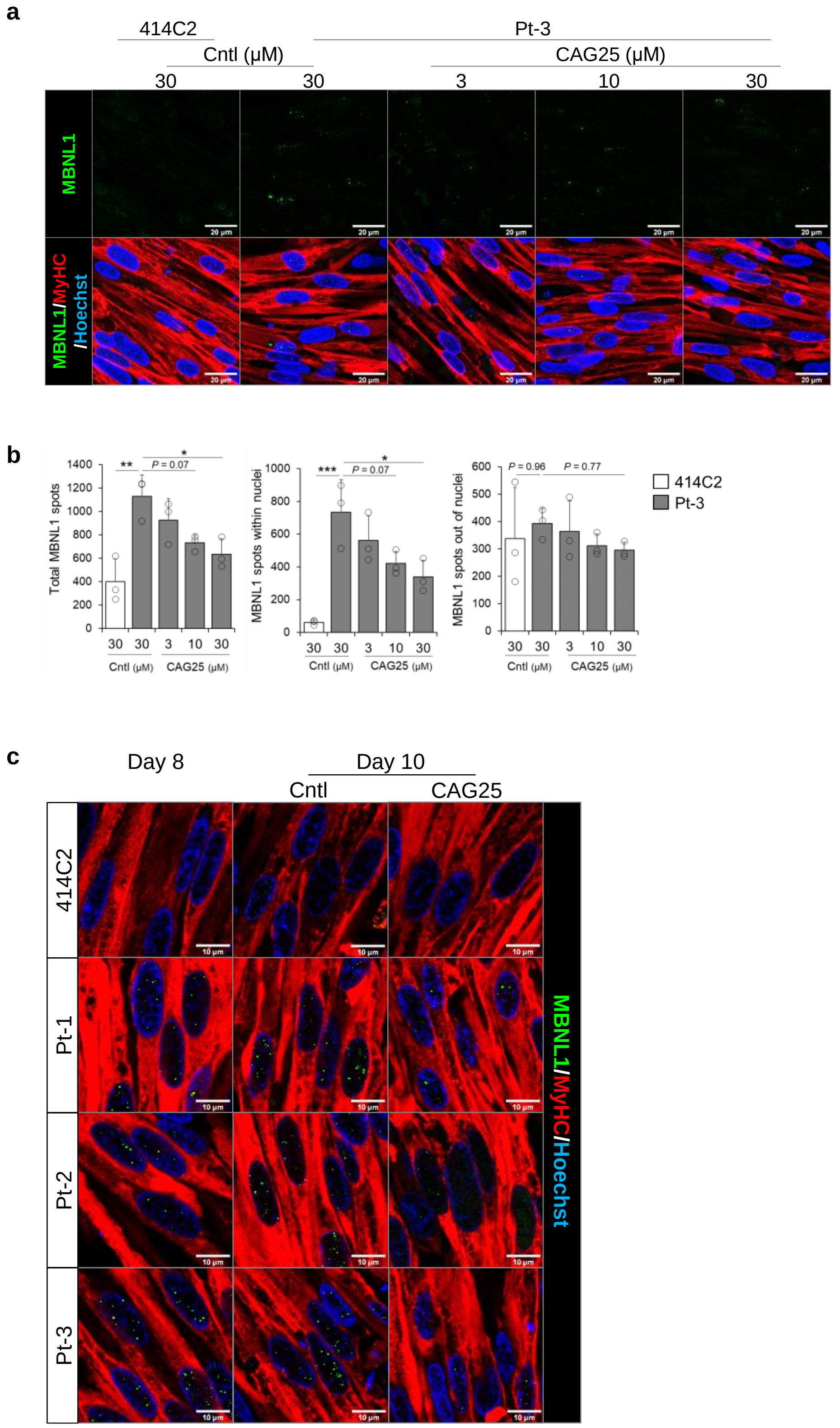

Supplementary Figure S6 Analysis of MBNL1 nuclear aggregation after CAG25 treatment in the MyoD1-induced system. Related to Figure 4

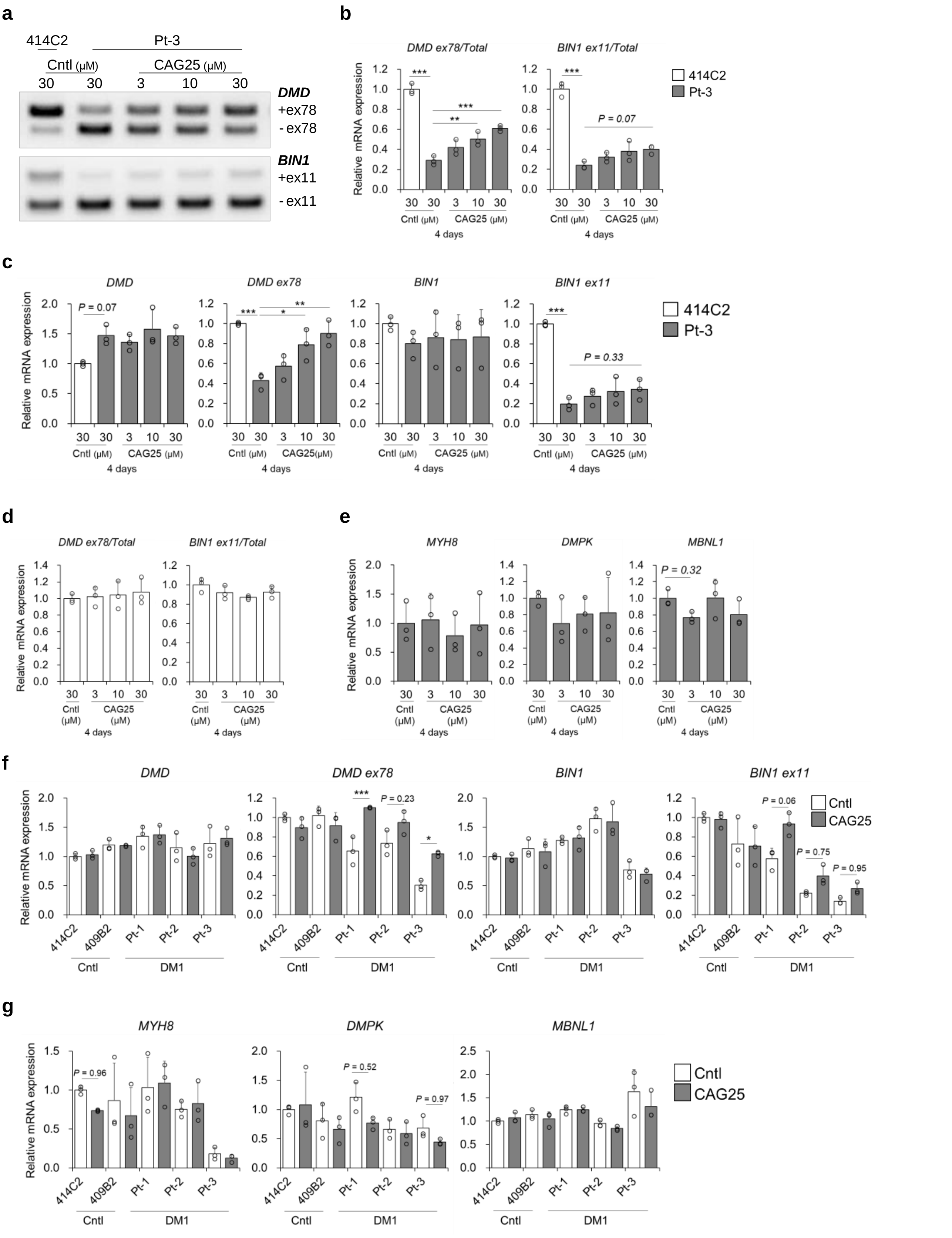

Supplementary Figure S7. Analysis of alternative splicing after CAG25 treatment in the MyoD1-induced system. Related to Figure 4

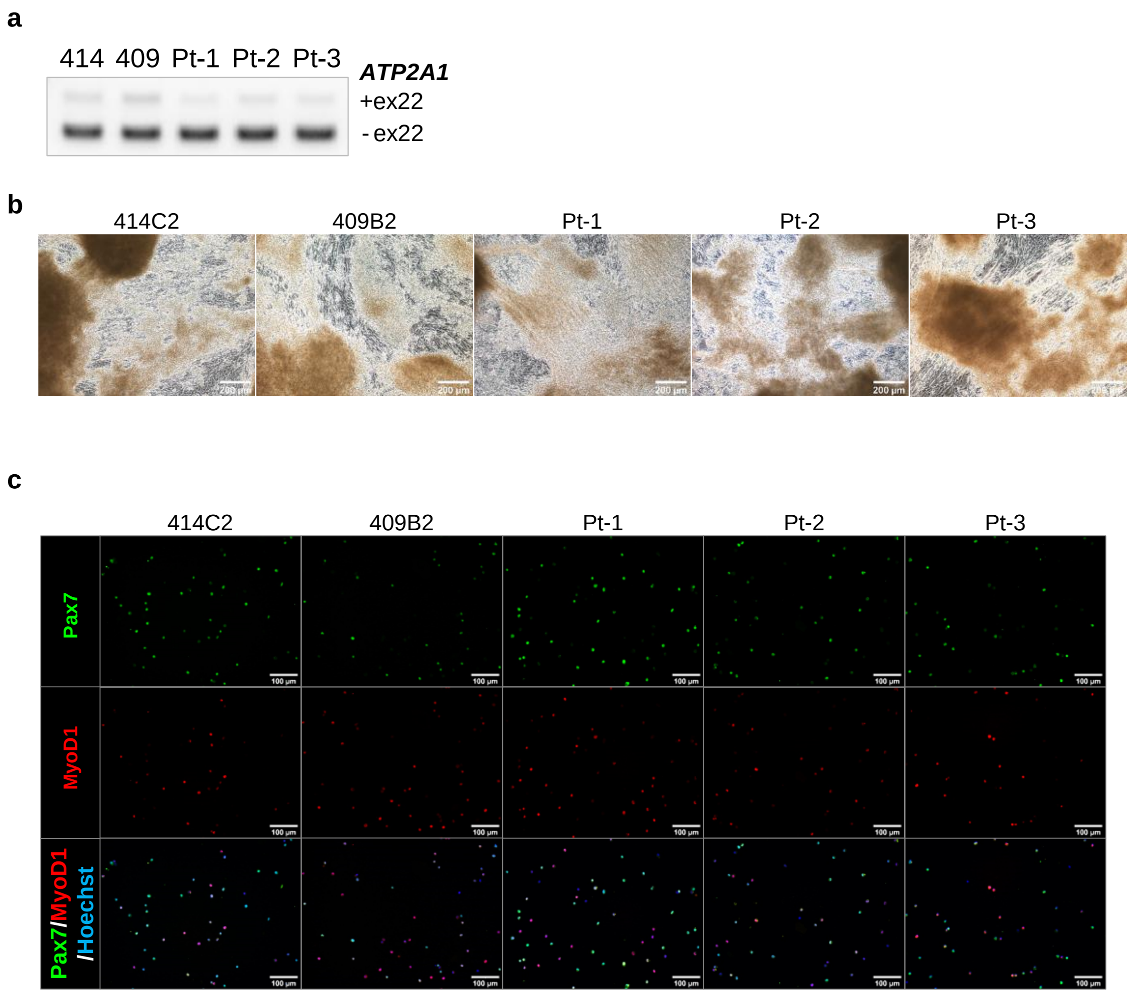

Supplementary Figure S8. Myogenic differentiation of hiPSCs using a stepwise protocol and protein expression of sorted CDH13-positive cells. Related to Figure 5

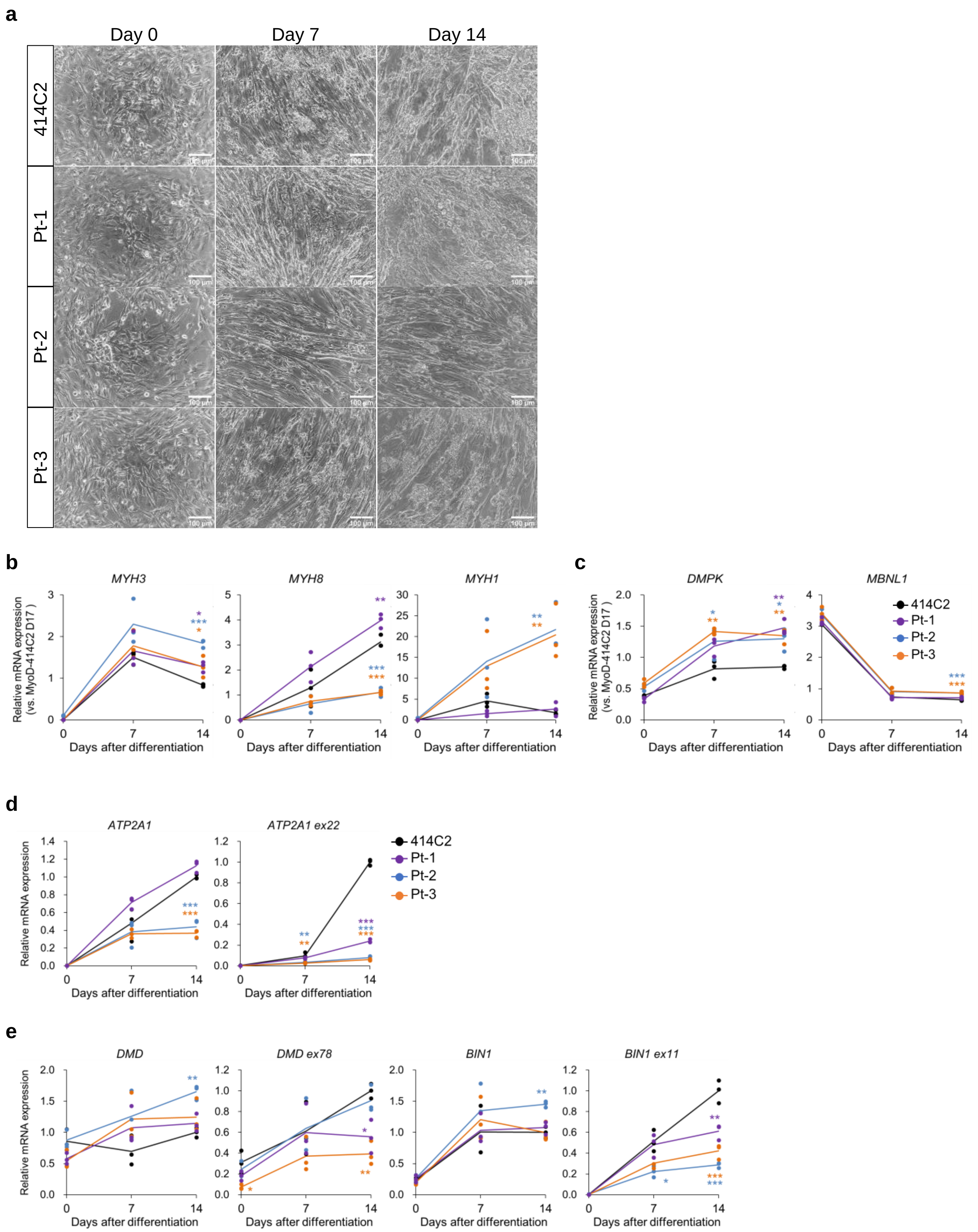

Supplementary Figure S9. Time-course analysis of gene expressions in the iMuSC differentiation system. Related to Figure 6

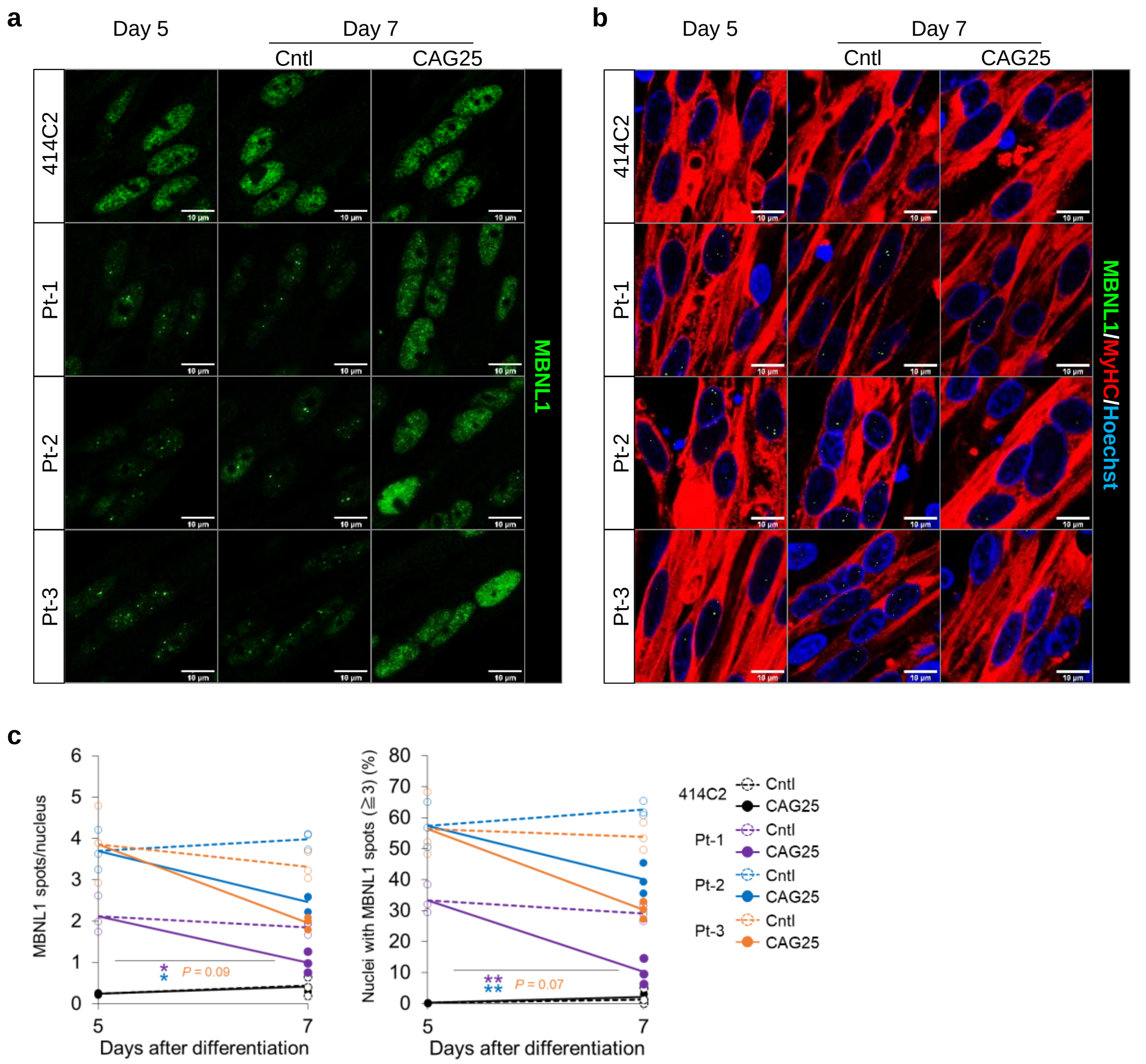

Supplementary Figure S10. Recovery of nuclear MBNL1 aggregation in DM1 cells by CAG25 treatment in the iMuSC differentiation system. Related to Figure 6

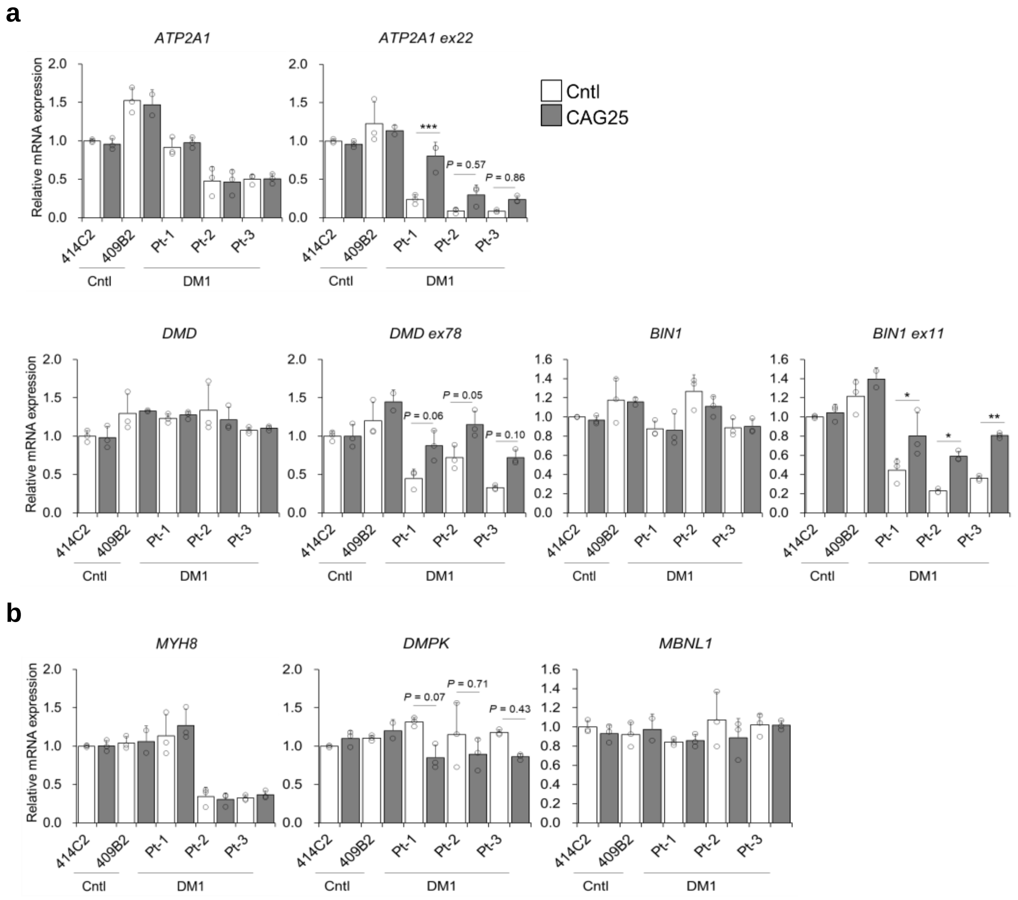

Supplementary Figure S11. Analysis of alternative splicing after CAG25 treatment in the iMuSC differentiation system. Related to Figure 6

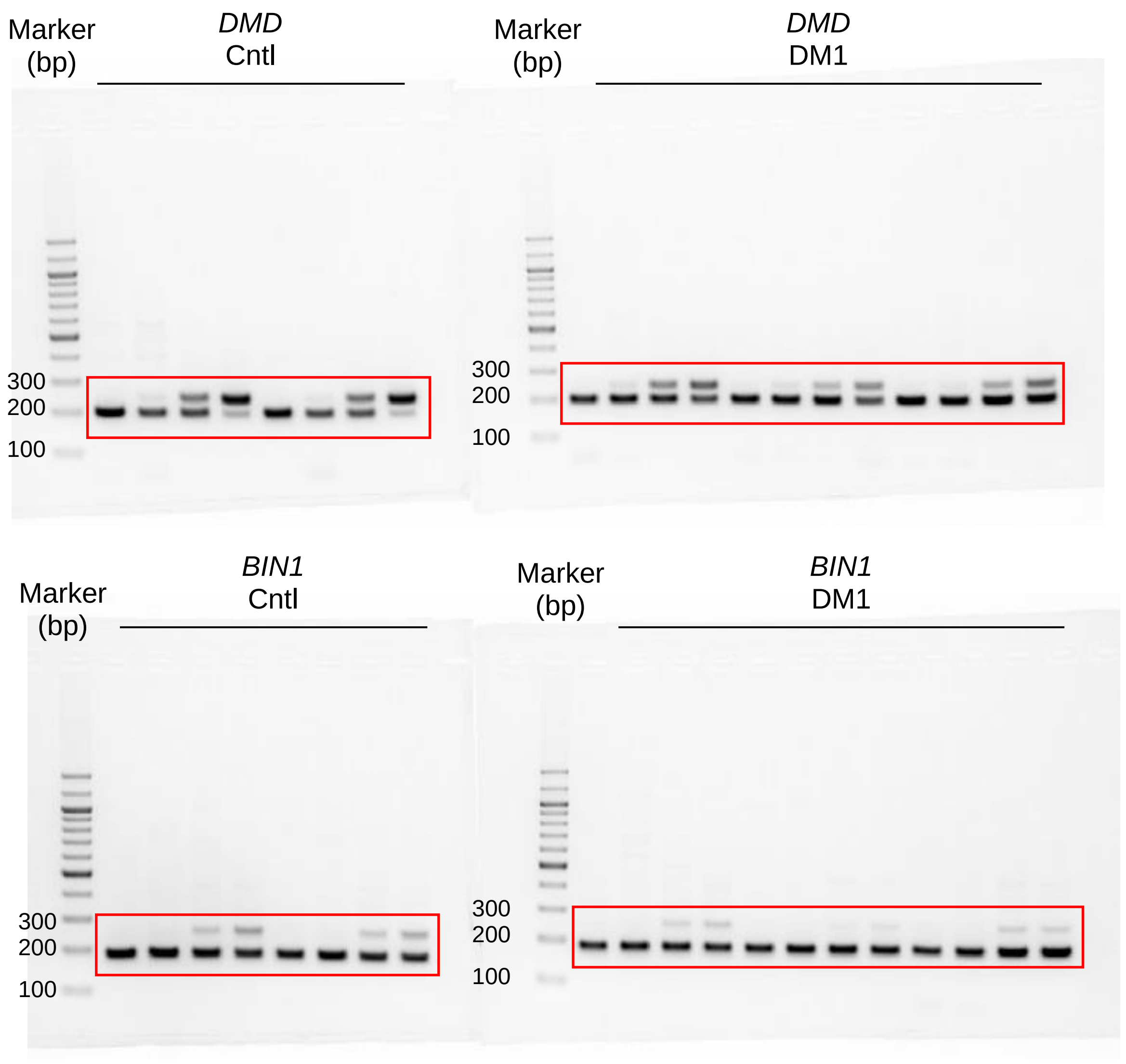

Supplementary Figure S12. Full-length gels presented in Figure 2g.

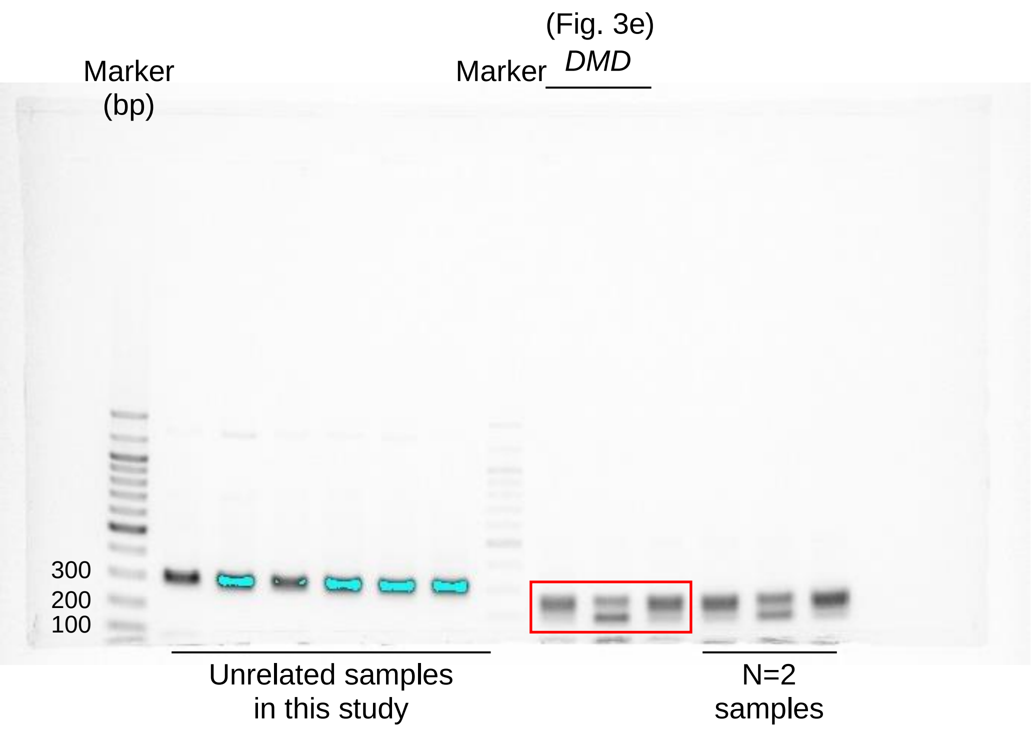

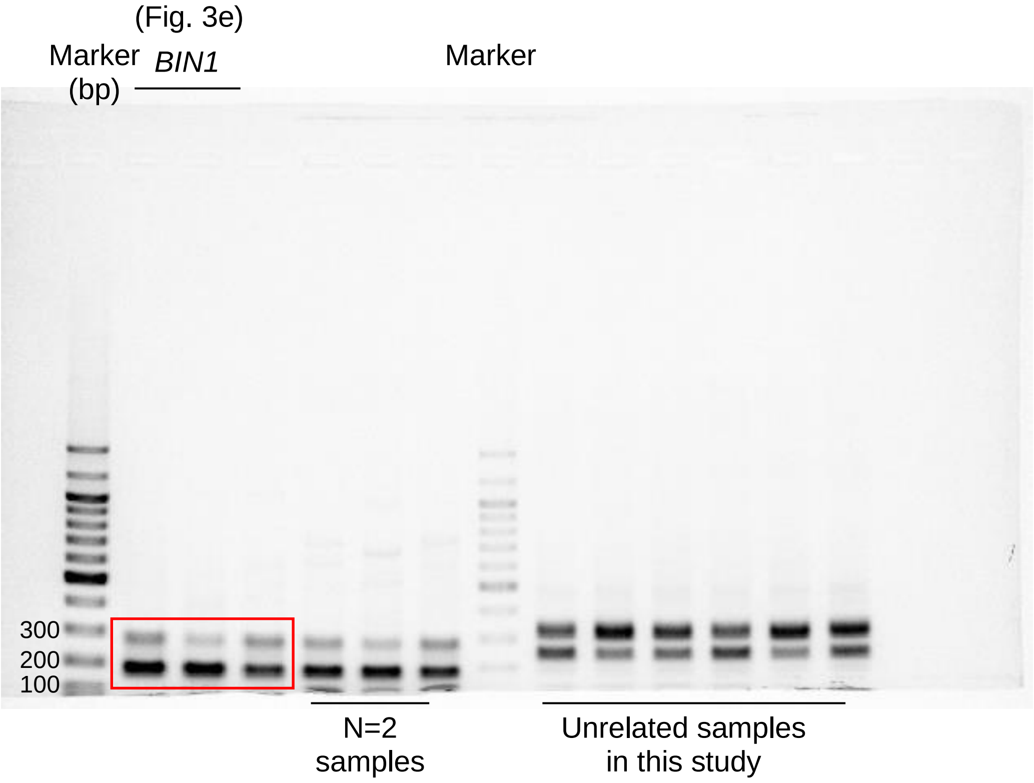

Supplementary Figure S13. Full-length gels presented in Figure 3e.

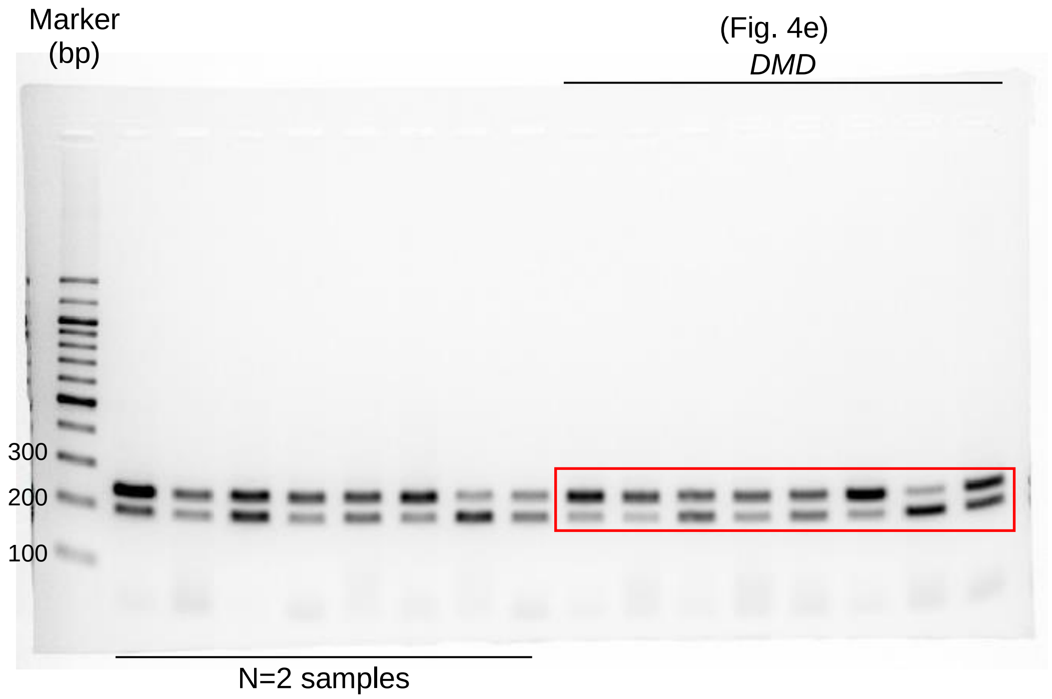

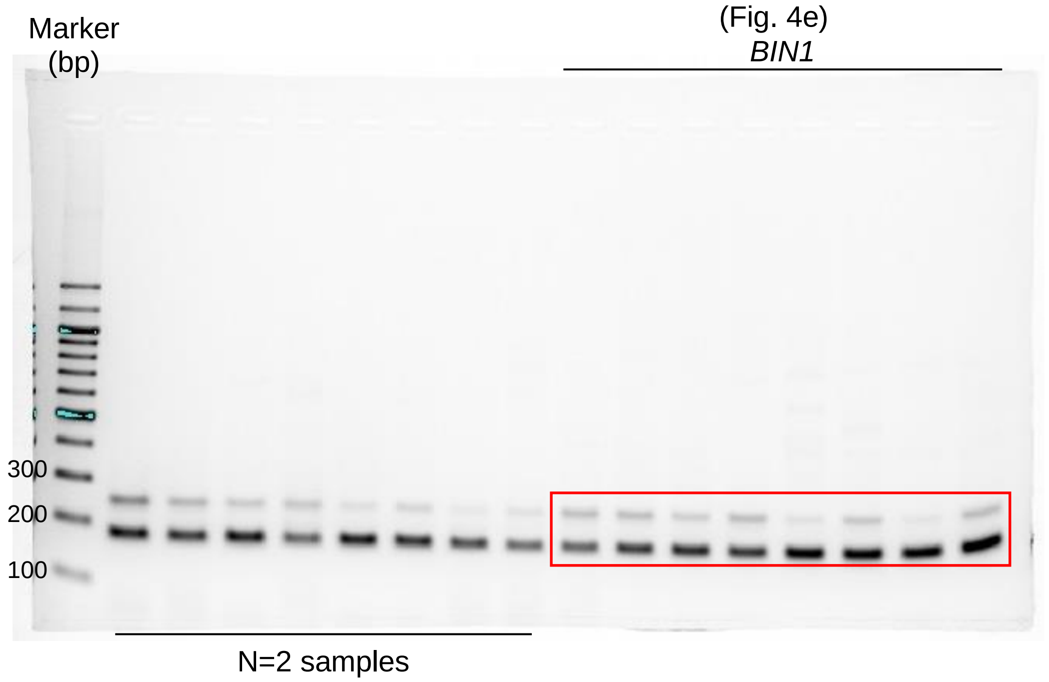

Supplementary Figure S14. Full-length gels presented in Figure 4e.

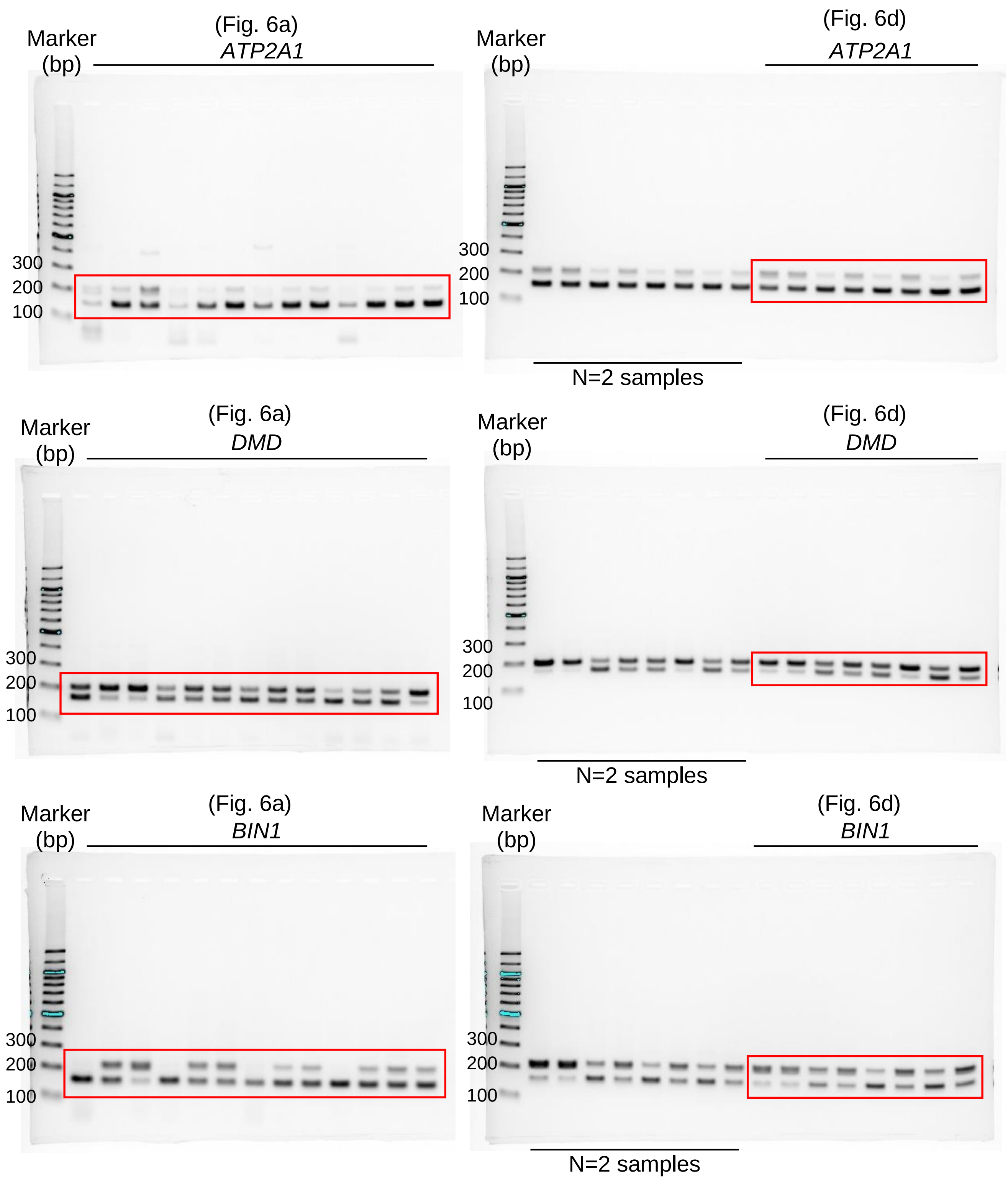

Supplementary Figure S15. Full-length gels presented in Fig. 6a and 6d.

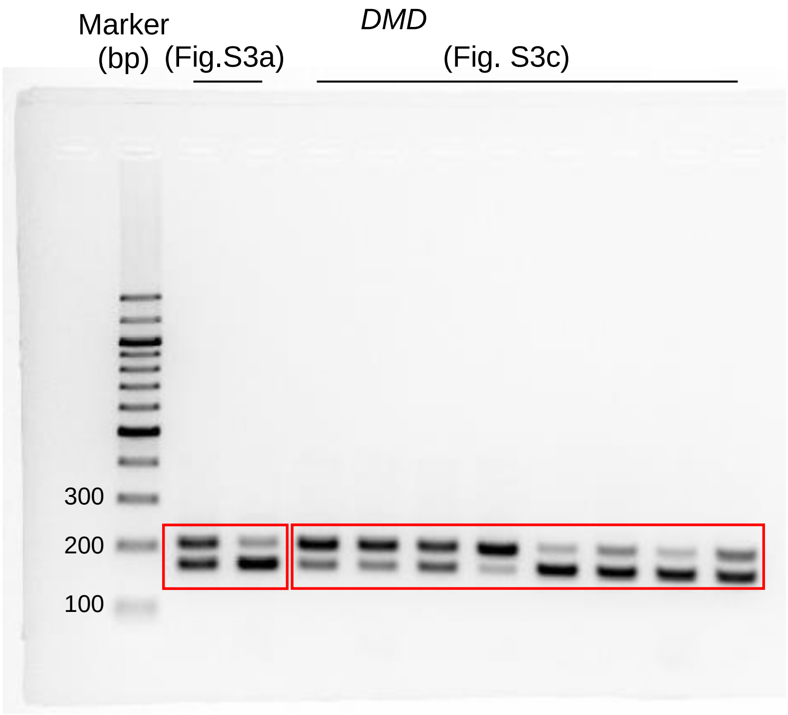

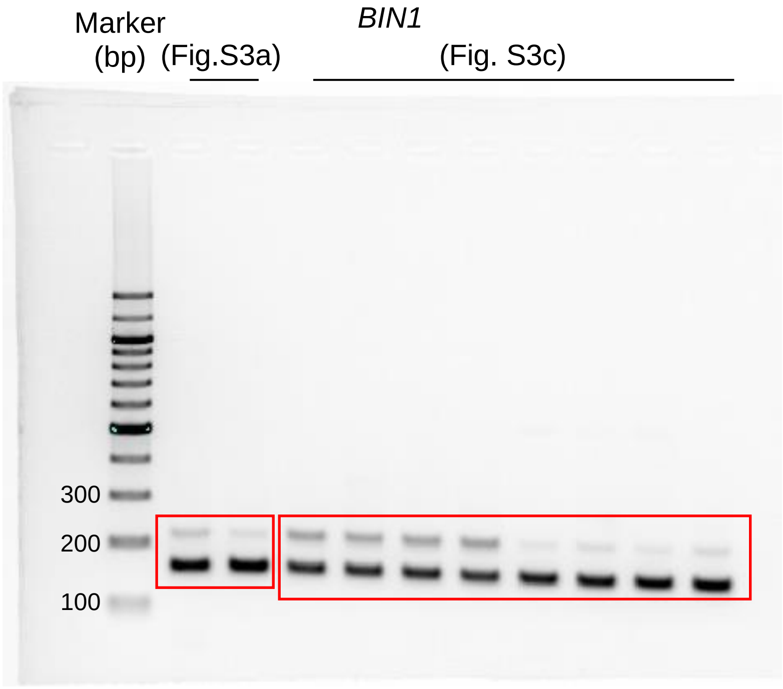

Supplementary Figure S16. Full-length gels presented in Fig. S3a and S3c.

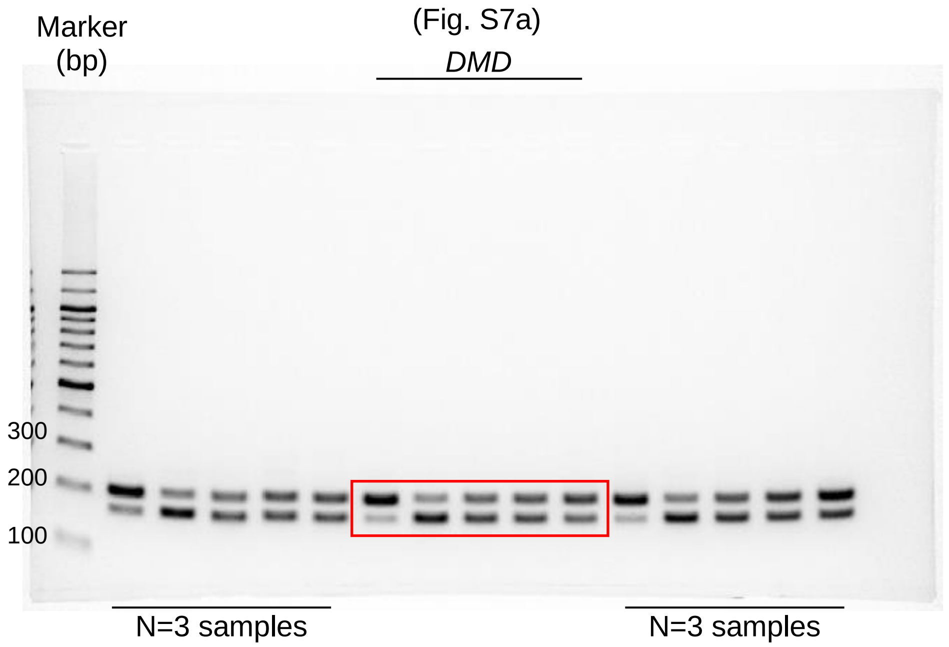

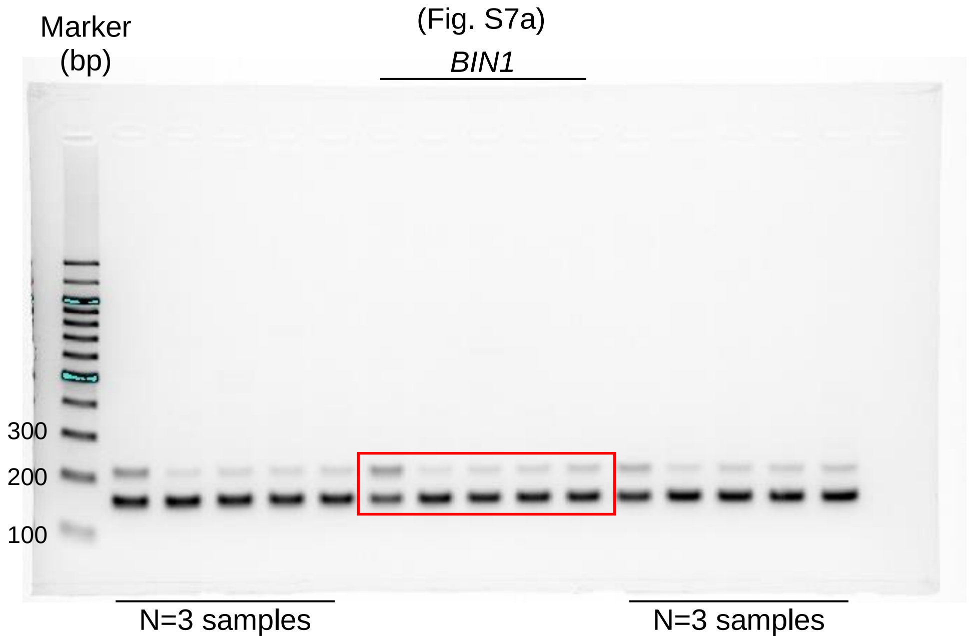

Supplementary Figure S17. Full-length gels presented in Fig. S7a.

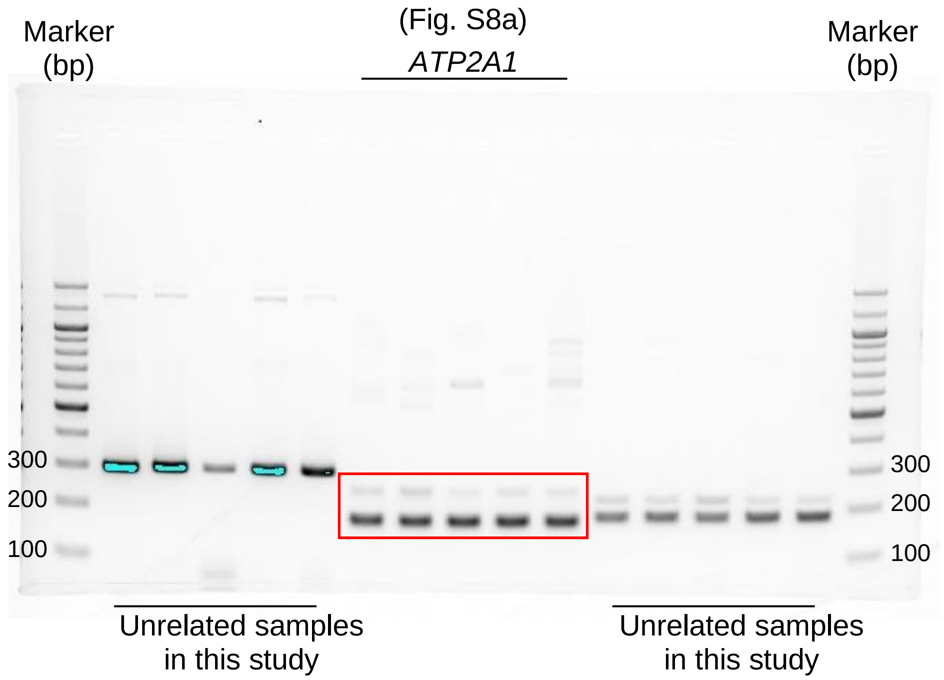

Supplementary Figure S18. Full-length gel presented in Fig. S8a.

ID	Sex	Age (years)	Source	Length of the CTG repeats at diagnosis	iPSC clone ID
Patient 1 (Pt-1)	Female	38	peripheral blood	900-1,100	CiRA00112
Patient 2 (Pt-2)	Female	1	dermal fibroblasts	>1,500	CiRA00211
Patient 3 (Pt-3)	Female	41	dermal fibroblasts	1,200-1,400	CIRA00213

Supplementary Table S1. Information of DM1-hiPSC lines

Antibody	Source	Host	Isotype	Dilution
Myosin Heavy chain (MyHC)	eBioscience, #14-6503 (MF20)	mouse, mono	IgG2b	1:800
MBNL1	DSHB, MB1a (4A8)	mouse, mono	IgG1	1:50
α-Actinin	Sigma, #A7811 (EA-53)	mouse, mono	IgG1	1:200
MyoD1	Abcam, #ab133627	rabbit, mono	IgG1	1:500
Pax7	Santacruz, #sc-81648	mouse, mono	IgG1κ	1:50
TRA-1-60	Millipore, #MAB4360	mouse, mono	IgM	1:100
CDH-13, APC-conjugated	Miltenyi Biotec, #130-111-264	mouse, mono	IgG1	1:200
Alexa Fluor 568 conjugated goat-anti-mouse IgG2b	Invitrogen, #A-21144	-	-	1:500
Alexa Fluor 488 conjugated goat-anti-mouse IgG1	Invitrogen, #A-21121	-	-	1:500
Alexa Fluor 568 conjugated goat-anti-rabbit IgG	Invitrogen, #A-11036	-	-	1:500
Alexa Fluor 488 conjugated goat-anti-mouse IgG	Invitrogen, #A-11034	-	-	1:500
Alexa Fluor 568 conjugated goat-anti-mouse IgM	Invitrogen, #A-21043	-	-	1:500

Supplementary Table S2. Antibody list

For Gel-based RT-PCR

Target gene	Forward sequence	Reverse Sequence
DMD ex78	GGTGATGGAGCAACTCAACAAC	AACCATGCGGGAATCAGGAG
BIN1 ex11	CTCAATGATGTGCTGGTCGG	GGCTCGTGGTTGACTCTGAT
ATP2A1 ex22	ATGGTCCTCAAGATCTCACTGC	AGCTCTGCCTGAAGATGTGTC

For Real-time RT-PCR

Target gene	Forward sequence	Reverse Sequence
CSNK2A2	AAAAGCTGCGACTGATAGATTGG	GAGGCTACACGAACATTGTACTC
OCT3/4	GACAGGGGGAGGGGAGGAGCTAGG	CTTCCCTCCAACCAGTTGCCCCAAAC
NANOG	CAGTCTGGACACTGGCTGAA	CTCGCTGATTAGGCTCCAAC
SOX2	GGGAAATGGGAGGGGTGCAAAAGAGG	TTGCGTGAGTGTGGATGGGATTGGTG
MYOD1	CACTCCGGTCCCAAATGTAG	TTCCCTGTAGCACCACACAC
MYOGENIN	TGGGCGTGTAAGGTGTGTAA	CGATGTACTGGATGGCACTG
MYH3	GCAGATTGAGCTGGAAAAGG	TCAGCTGCTCGATCTCTTCA
MYH8	AATGCAAGTGCTATTCCAGAGG	ACAGACAGCTTGTGTTCTTGTT
MYH1	CTGGTGGACAAACTGCAAGC	TGGATCCTGCGGAATTTGGAG
DMPK	GGCCAGGTGTATGCCATGAA	CCGCCCACGTAATACTCCA
MBNL1	GGACGAGTAATCGCCTGCTTT	TCTGCTGAATCAAGTTATTGCGT
PAX7	AGGGCCTCCTGCTTGTTTAT	GGTTTTGCCCAACTCAGTGT
MYF5	TCACCTCCTCAGAGCAACCT	GGAACTAGAAGCCCCTGGAG
DMD (Total)	GAGTGGTTGGCAGTCAAACTTC	GTTGTTGAGTTGCTCCATCACC
DMD ex78	GGTGATGGAGCAACTCAACAAC	TGTCCTCTCTCATTGGCTTTCC
BIN1 (Total)	TGAGCAGTGCGTCCAGAATTT	CGATCTTGTTTGCCTCATCCC
BIN1 ex11	AATGATGTGCTGGTCGGCCT	GTTCTTCTTTCTGCGCAGCC
ATP2A1 (Total)	CTGTGTGGCTGTCTGGCTTA	CAGGAAGACCTTCGGGGATG
ATP2A1 ex22	TCAAGTTCGTTGCTCGGAAC	GTGACACGGGCTCAGAGATG

Supplementary Table S3. Primer sets list for RT-PCR

Supplementary Figure Legends

Supplementary Figure S1. Colocalization of nuclear MBNL1 aggregation and CUGexp foci in the myotubes differentiated from MyoD-DM1-hiPSC. Related to Figure 1

(a) Time-course of the mRNA expressions of the myogenic markers, *MYOD1* and *MYOGENIN*, and the embryonic skeletal muscle marker, *MYH3,* by RT-qPCR analyses on day 0, day 3 and day 10 of differentiation. The gene expressions were indicated by relative values to 414C2 control on day 10. The data represent the means and were analyzed by one-way ANOVA followed by Tukey's test from three independent experiments (**P* < 0.05, ***P* < 0.01, vs. 414C2 control and # *P* < 0.05, ## *P* < 0.01, vs. 409B2 control). (b) Representative images of combined FISH-immunohistochemistry using Cy3-(CAG)7 probe and anti-MBNL1 antibody after PFA or acetone-MeOH fixation on day 10. Scale bars, 10 μm.

Supplementary Figure S2. Time-course analysis of nuclear MBNL1 aggregation in MyoD-hiPSCs. Related to Figure 1

(a-c) Time-course evaluation of nuclear MBNL1 aggregation after myogenic differentiation. Representative immunohistochemistry images after (a) PFA fixation and (b) acetone-MeOH fixation for MBNL1, TRA1-60, MyHC and Hoechst on day 0, day 4 and day 10. Arrowheads show nuclear MBNL1 aggregates in acetone-MeOH fixed cells. Scale bars, 20 μm. (c) Quantitative analyses of MBNL1 immunostaining images after acetone-MeOH fixation, using the ImageJ software. The nuclear regions were detected by Hoechst counterstaining. Data represent the means from three independent experiments and were analyzed by one-way ANOVA followed by Tukey's test (****P* < 0.001, vs. 414C2 control).

Supplementary Figure S3. Modification of the MyoD1-induced system to recapitulate the splicing defects in the DM1 cells. Related to Figure 2

(a) Representative images of alternative splicing analysis for *DMD* and *BIN1* by gel-based RT-PCR on day 10. The target exons are indicated on the right. (b) The mRNA expressions of *MYH8*, *DMD* exon 78 and *BIN1* exon 11 by RT-qPCR analyses in the 414C2 and Pt-3 myotubes on day 17. The gene expressions are indicated by relative values to the DMEM+Ara-C group in each cell line. Data represent the means+SD and were analyzed by two-way ANOVA followed by Tukey's test from three independent experiments (**P* < 0.05, ***P* < 0.01, ****P* < 0.001). (c) Representative images of alternative splicing analysis for *DMD* and *BIN1* by gel-based RT-PCR on day 17. (d, e) Evaluation of MyoD1-induced myogenic differentiation efficiency in 409B2, Pt-1 and Pt-2 myotubes on day 17. (d) Representative images of immunohistochemistry for MyHC and Hoechst. Scale bars, 500 μm. (e) Quantitative analyses of the MyHC-positive area using a BZ-X analyzer software. Data represent the means+SD from three independent experiments and were analyzed by two-way ANOVA followed by Tukey's test (**P* < 0.05, ****P* < 0.001).

Supplementary Figure S4. Time-course analysis of gene expressions with the use of the newly

modified protocol for MyoD1-induced system. Related to Figure 2

(a) Phase contrast images of the time-course of myogenic differentiation. Scale bars, 100 μm. (b-e) Time-course mRNA expressions of the (b) pluripotency markers, *OCT3/4*, *NANOG* and *SOX2*, (c) DM1-related genes, *DMPK* and *MBNL1*, (d) myogenic markers, *MYOD1* and *MYOGENIN*, and myosin heavy chains, *MYH3* and *MYH8* and (e) alternative splicing for *DMD* and *BIN1* by RT-qPCR analyses on day 0, day 3, day 10 and day 17 of the differentiation. The gene expressions are indicated by relative values to 414C2 control on day 0 or day 17. Data represent the means three independent experiments and were analyzed by one-way ANOVA followed by Tukey's test (*$P < 0.05$, **$P < 0.01$, ***$P < 0.001$, vs. 414C2 control and # $P < 0.05$, ## $P < 0.01$, ### $P < 0.001$, vs. 409B2 control).

Supplementary Figure S5. Analyses of DM1 phenotypes using CTGexp-deleted MyoD-DM1-hiPSCs. Related to Figure 3

(a and b) Establishment of the CTGexp-deleted hiPSC line. (a) Image of agarose gel electrophoresis after genomic PCR of the DNA samples extracted from the 414C2 control, Pt-1 and genome-edited Pt-1 cells. Long amplicons of over 5k base pairs (bp) in the Pt-1 cells was abolished in the Pt-1ΔCTG cells (b) The sequencing results of the DNA extract from the agarose gels (the predicted amplicon length: 497 bp and 370 bp) aligned with the 3' untranslated region (UTR) of *DMPK* sequence (NM_001081563). Sequencing of the extract from the 414C2 band (1), Pt-1 band (2), the Pt-1ΔCTG band, long one (3) and Pt-1ΔCTG, short one (4) are shown. Sequencing of the ΔCTG short band (4) showed that the CTG repeats region around two gRNAs was excised out by Cas9, and a partial deletion of CTG repeats in a unilateral allele was observed by analysis of the ΔCTG long band. (c) Representative images of immunohistochemical staining after acetone-MeOH fixation for MBNL1, MyHC and Hoechst on day 10 of differentiation. Scale bars, 10 μm. (d, e) mRNA expressions of (d) alternative splicing for *DMD* and *BIN1* and (e) *MYH8*, *DMPK* and *MBNL1* by RT-qPCR analyses on day 17. The gene expressions are indicated by relative values to 414C2 control. The data represent the means of three independent experiments and were analyzed by one-way ANOVA, followed by Tukey's test (*$P < 0.05$, **$P < 0.01$, ***$P < 0.001$).

Supplementary Figure S6. Analysis of MBNL1 nuclear aggregation after CAG25 treatment in the MyoD1-induced system. Related to Figure 4

(a and b) Evaluation of nuclear MBNL1 aggregation after two days of CAG25 treatment in 414C2 and Pt-3 myotubes on day 10. (a) Representative images of immunohistochemical staining after acetone-MeOH fixation for MBNL1, MyHC and Hoechst. Scale bars, 20 μm. (b) Further quantitative analyses of the images of MBNL1 immunostaining after acetone-MeOH fixation using the ImageJ software. The nuclear regions were detected by Hoechst counterstaining. The data represent the means+SD of three independent experiments and were analyzed by two-way ANOVA, followed by Tukey's test (*$P < 0.05$, **$P < 0.01$, ***$P < 0.001$). (c) The representative images by immunohistochemistry after acetone-MeOH fixation for MBNL1, MyHC and Hoechst on day 8 and 10. The cells were treated with CAG25 from day 8 to day 10. Scale bars,

10 μm.

Supplementary Figure S7. Analysis of alternative splicing after CAG25 treatment in the MyoD1-induced system. Related to Figure 4

(a-e) Evaluation of alternative splicing for *DMD* and *BIN1* after four days of CAG25 treatment in the 414C2 and Pt-3 myotubes on day 17. (a) Representative images obtained by gel-based RT-PCR. The target exons are indicated on the right. (b-d) The results of quantitative analyses of alternative splicing for *DMD* and *BIN1* by RT-qPCR. (e) The mRNA expressions of *MYH8*, *DMPK* and *MBNL1* by RT-qPCR analyses in the Pt-3 myotubes. (f and g) Evaluation of alternative splicing for *DMD* and *BIN1* after four days of CAG25 treatment in the 414C2, 409B2, Pt-1, Pt-2, and Pt-3 myotubes on day 17. (f) The results of quantitative analyses of alternative splicing for *DMD* and *BIN1* exons by RT-qPCR. (g) The mRNA expressions of *MYH8*, *DMPK* and MBNL1 by RT-qPCR analyses. The gene expressions are indicated by relative values to 414C2 control treated with control oligonucleotide. The data represent the means+SD from three independent experiments and were analyzed by two-way ANOVA, followed by Tukey's test (*$P < 0.05$, **$P < 0.01$, ***$P < 0.001$).

Supplementary Figure S8. Myogenic differentiation of hiPSCs using a stepwise protocol and protein expression of sorted CDH13-positive cells. Related to Figure 5

(a) Evaluation of alternative splicing for *ATP2A1* in MyoD1-mediated myotubes on day 17. Representative images obtained by gel-based RT-PCR. The target exons are indicated on the right. (b) Phase-contrast images of myogenic differentiation at week 12-13 by a stepwise protocol. Scale bars, 200 μm. (c) Representative images of immunohistochemical staining for Pax7, MyoD1 and Hoechst. Scale bars, 100 μm.

Supplementary Figure S9. Time-course analysis of gene expressions in the iMuSC differentiation system. Related to Figure 6

(a) Phase-contrast images of the time-course of iMuSC in vitro myogenic differentiation. Scale bars, 100 μm. (b-e) Time-course of mRNA expressions of the (b) myosin heavy chains, *MYH3*, *MYH8* and *MYH1*, (c) DM1-related genes, *DMPK* and *MBNL1*, (d) alternative splicing for *ATP2A1* (e) alternative splicing for *DMD* and *BIN1* by RT-qPCR analyses on day 0, day 7 and day 14 of iMuSC differentiation. The gene expressions are indicated by relative values to MyoD-414C2 control on day 17 (b and c) or iMuSC-414C2 control on day 14 (d and e). The data represent the means of three independent experiments and were analyzed by one-way ANOVA, followed by Tukey's test (*$P < 0.05$, **$P < 0.01$, ***$P < 0.001$, vs. 414C2 control).

Supplementary Figure S10. Recovery of nuclear MBNL1 aggregation in DM1 cells by CAG25 treatment in the iMuSC differentiation system. Related to Figure 6

(a-c) Evaluation of nuclear MBNL1 aggregation after two days of CAG25 treatment in DM1 myotubes on day 7 of iMuSC differentiation. (a and b) Representative immunohistochemistry images after (a) PFA fixation or (b) acetone-MeOH fixation for MBNL1, MyHC and Hoechst. Scale bars, 10 μm. (c) Quantitative analyses

of images of MBNL1 immunostaining after acetone-MeOH fixation. The data represent the means of three independent experiments and were analyzed by the paired t-test (*$P < 0.05$, **$P < 0.01$).

Supplementary Figure S11. Analysis of alternative splicing after CAG25 treatment in the iMuSC differentiation system. Related to Figure 6

(a and b) Evaluation on day 14 of alternative splicing for *ATP2A1*, *DMD* and *BIN1* after seven days of CAG25 treatment. (a) The results of quantitative analyses of alternative splicing by RT-qPCR. (b) The mRNA expressions of *MYH8*, *DMPK* and *MBNL1* by RT-qPCR analyses. The gene expressions are indicated by relative values to 414C2 control treated with control oligonucleotide. The data represent the means+SD and were analyzed by two-way ANOVA, followed by Tukey's test from three independent experiments (*$P < 0.05$, **$P < 0.01$, ***$P < 0.001$).

Supplementary Figure S12. Full-length gels presented in Figure 2g.

Red boxes indicate the cropped regions of the original full-length gels used in the main figure.

Supplementary Figure S13. Full-length gels presented in Figure 3e.

Red boxes indicate the cropped regions of the original full-length gels used in the main figure.

Supplementary Figure S14. Full-length gels presented in Figure 4e.

Red boxes indicate the cropped regions of the original full-length gels used in the main figure.

Supplementary Figure S15. Full-length gels presented in Fig. 6a and 6d.

Red boxes indicate the cropped regions of the original full-length gels used in the main figure.

Supplementary Figure S16. Full-length gels presented in Fig. S3a and S3c.

Red boxes indicate the cropped regions of the original full-length gels used in the supplementary figure.

Supplementary Figure S17. Full-length gels presented in Fig. S7a.

Red boxes indicate the cropped regions of the original full-length gels used in the supplementary figure.

Supplementary Figure S18. Full-length gel presented in Fig. S8a.

Red box indicates the cropped region of the original full-length gel used in the supplementary figure.

Supplementary Table S1. Information of DM1-hiPSC lines

Supplementary Table S2. Antibody list

Supplementary Table S3. Primer sets list for RT-PCR
